# Contribution of Stochastic Partitioning at Human Embryonic Stem Cell Division to NANOG Heterogeneity

**DOI:** 10.1371/journal.pone.0050715

**Published:** 2012-11-30

**Authors:** Jincheng Wu, Emmanuel S. Tzanakakis

**Affiliations:** 1 Department of Chemical and Biological Engineering, State University of New York at Buffalo, Buffalo, New York, United States of America; 2 Department of Biomedical Engineering, State University of New York at Buffalo, Buffalo, New York, United States of America; 3 New York State Center of Excellence in Bioinformatics and Life Sciences, State University of New York at Buffalo, Buffalo, New York, United States of America; 4 Western New York Stem Cell Culture and Analysis Center, State University of New York at Buffalo, Buffalo, New York, United States of America; University of Newcastle upon Tyne, United Kingdom

## Abstract

Heterogeneity is an often unappreciated characteristic of stem cell populations yet its importance in fate determination is becoming increasingly evident. Although gene expression noise has received greater attention as a source of non-genetic heterogeneity, the effects of stochastic partitioning of cellular material during mitosis on population variability have not been researched to date. We examined self-renewing human embryonic stem cells (hESCs), which typically exhibit a dispersed distribution of the pluripotency marker NANOG. In conjunction with our experiments, a multiscale cell population balance equation (PBE) model was constructed accounting for transcriptional noise and stochastic partitioning at division as sources of population heterogeneity. Cultured hESCs maintained time-invariant profiles of size and NANOG expression and the data were utilized for parameter estimation. Contributions from both sources considered in this study were significant on the NANOG profile, although elimination of the gene expression noise resulted in greater changes in the dispersion of the NANOG distribution. Moreover, blocking of division by treating hESCs with nocodazole or colcemid led to a 39% increase in the average NANOG content and over 68% of the cells had higher NANOG level than the mean NANOG expression of untreated cells. Model predictions, which were in excellent agreement with these findings, revealed that stochastic partitioning accounted for 17% of the total noise in the NANOG profile of self-renewing hESCs. The computational framework developed in this study will aid in gaining a deeper understanding of how pluripotent stem/progenitor cells orchestrate processes such as gene expression and proliferation for maintaining their pluripotency or differentiating along particular lineages. Such models will be essential in designing and optimizing efficient differentiation strategies and bioprocesses for the production of therapeutically suitable stem cell progeny.

## Introduction

Human pluripotent stem cells (hPSCs) including embryonic (hESCs) and induced pluripotent stem cells (hiPSCs) self-renew extensively and under appropriate conditions give rise to multiple cell types. These properties make hPSCs invaluable both as tools for studying development and as a source of therapeutics for regenerative medicine. The switch between self-renewal and differentiation as well as commitment along a particular lineage are often thought as a series of choices between binary alternate states mediated by coordinated actions at multiple levels, i.e. from gene networks to extracellular factor-activated signaling cascades [1], [2].

Nevertheless, a commonly observed but unappreciated attribute of stem cell ensembles in vivo/vitro is their heterogeneity. Cells in the inner cell mass of mouse blastocysts express Oct4, Nanog and Gata6 in a mutually exclusive and seemingly random ‘salt-and-pepper’ pattern [3] depending on extracellularly-induced signaling cascades. Cultured ESCs also exhibit inhomogeneous expression of POU5F1 (Oct4), Nanog, SSEA1, SSEA3, Stella and Rex1 [4], [5], [6], [7], [8], [9], [10]. Heterogeneity is also noted in other stem/progenitor cells including neural [11], intestinal [12], [13] and hematopoietic stem cells (HSCs) [14]. Hence, heterogeneity is a characteristic of stem/progenitor cell populations affecting their ability to self-renew and differentiate but its exact physiological role(s) remains unclear. For instance, the heterogeneous expression of genes from genetically identical hESCs has been linked to lineage primed subpopulations co-expressing pluripotency and lineage-specific markers. Heterogeneity may also underlie the variable response of stem cells to differentiation cues resulting in particular tissue patterns.

Nanog is a key pluripotency regulator that shows relatively lower expression levels and more significant heterogeneity among hESC populations than other core stemness transcription factors such as OCT4 and SOX2 [15], [16], [17], [18]. For instance, ∼20% of mouse ESCs (mESCs) have no detectable expression of Nanog (Nanog^−^) and despite their expression Oct4 and SSEA1 [5] they can reconstitute the original mESC population including Nanog^+^ cells. The downregulation or transient depletion of Nanog is linked to loss of pluripotency and commitment [5], [19], [20] whereas its overexpression prevents ESCs from differentiating. Then, sources of Nanog variability conceivably influence the balance between self-renewal and differentiation. To date, Nanog heterogeneity has been attributed to stochasticity in its gene expression. A transcriptional noise-driven excitable system featuring a feedback loop with Oct4 (gene regulatory network) was constructed to describe the dynamics of Nanog expression in mESCs [21]. The model reveals noise-induced excursions from a Nanog^high^ to a Nanog^low^ state in which the cells are prone to differentiate in the presence of appropriate cues. Whether the time it takes for cells to transition between the two states is adequate to explain the residence of cells in the latter state is an open question. Alternative scenarios of Nanog regulation through fluctuations or oscillations of its expression have also been investigated [22].

Although stochastic gene expression has received increased attention, the emergence of heterogeneous cell populations is also influenced by other processes, notably cell division and the associated stochastic partitioning of cellular material [23], [24], [25]. Each of the daughter cells generated from a mESC expressing GFP from the *Nanog* locus exhibits different Nanog levels based on GFP fluorescence [21]. Cell mitosis may contribute to the stochastic fluctuations of Nanog as it inevitably leads to partitioning of the Nanog mRNA/protein and factors (e.g. transcription factors) pertinent to its synthesis and degradation in the two newborn cells. Therefore, Nanog levels in hPSCs may be regulated through the interplay between Nanog mRNA/protein expression, degradation and dilution due to mitosis. In fact, cell division (and thus partitioning) and protein degradation have comparable effects especially in moderately proliferating cells [23] influencing protein levels along with gene expression. Interestingly enough, the G_1_ phase is a critical window for the differentiation of hESCs exposed to proper signals [26], [27], [28]. Consequently, the decision of hESCs to select a particular fate trajectory may be more sensitive to the fluctuation of Nanog immediately after division. This is also consistent with the support of hESC self-renewal by a shortened G_1_ phase preventing loss of pluripotency [29]. Furthermore, overexpression of Nanog results in faster growth of hESCs and shorter time for S-phase entry [30]. These findings link the maintenance of pluripotency, Nanog level and cell cycle dynamics and point to a universal role of cell division in stem cell fate selection processes.

In this study we investigated the heterogeneity in Nanog expression of self-renewing hESC populations arising from stochasticity in gene expression and partitioning at cell mitosis. For this purpose, a population balance equation (PBE)-based quantitative framework was developed for pluripotent hESCs taking into account these two sources of heterogeneity. The state vector comprised hESC size and Nanog expression as descriptors of proliferation and pluripotency, respectively. The model was coupled to experimental observations and parameters were determined via Monte Carlo (MC) simulations. Subsequently, the NANOG profile was predicted for growth-arrested hESCs and the results were compared to measurements in hESC populations treated with anti-mitosis agents. More importantly, the relative contributions of cell partitioning and gene expression noise to the expression dynamics of Nanog in self-renewing hESCs were quantified for the first time.

## Materials and Methods

### Human Embryonic Stem Cell Culture

The hESC line H9 (passages 30–60) was obtained from the WiCell Research Institute (Madison, WI) and its use was approved by the Committee for Stem Cell Research Oversight at SUNY-Buffalo. Cells were cultured in dishes coated with Matrigel (BD Biosciences, San Jose, CA) and in mTeSR1 medium (StemCell Technologies, Vancouver, BC). The cultures were maintained in 5% CO_2_/95% air at 37°C. Medium was replaced every day, and the cells were passaged every 5–6 days by enzymatic dissociation with collagenase type IV (GIBCO, Grand Island, NY).

Cultured cell viability was assessed by Trypan Blue staining (Sigma-Aldrich, St. Louis, MO) and counting in a hemocytometer. Alternatively, cells were stained with 20 mg/ml fluorescein diacetate (FDA-live cells; Sigma-Aldrich) in PBS for 5 min and after being washed twice with PBS, they were analyzed by flow cytometry. Karyotypic analysis of cultured hESCs is routinely performed at the SKY/FISH facility at Roswell Park Cancer Institute (Buffalo, NY).

### Flow Cytometry

Colonies on Matrigel-coated dishes were dissociated into single cells with Accutase (Innovative Cell Technologies Inc., San Diego, CA) and collected by centrifugation at 200×g for 5 min. Cells were fixed for 10 min in 3.7% formaldehyde solution (Sigma-Aldrich, St. Louis, MO), washed with PBS (Mediatech Inc., Manassas, VA) and blocked with 3% normal donkey serum (Jackson ImmunoResearch Laboratories, West Grove, PA) for 30 min before incubation with a monoclonal PE-conjugated mouse anti-human NANOG antibody (cat. no. 560483) or an isotype control (cat. no. 554680; both from BD Biosciences, Franklin Lakes, NJ) for 1 hr at room temperature. The specificity of the NANOG antibody was tested and verified in hESCs, differentiated hESCs and non-stem cells (Fig. S1). Sample analysis was carried out on a FACS Calibur flow cytometer with the CellQuest software (BD Biosciences). Data were further analyzed with the FCS Express V4.0 suite (De Novo Software, Los Angeles, CA). Cells were registered as positive if their emitted fluorescence level was above 98% of that of isotype control samples. Quantum PE MESF (Molecules of Equivalent Soluble Fluorochrome intensity) beads (Bangs Laboratories, Fishers, IN) were used for calibration [31] and conversion of relative fluorescence intensities to MESF units and FSC data to cell size (Fig. S2).

### Growth Arrest Experiments and Cell Cycle Analysis

Four to five days after plating, cells were treated with 200 ng/ml nocodazole (Sigma-Aldrich) or 100 ng/ml colcemid (GIBCO) for 12–20 hr [29] as stated. Subsequently, the cells were washed twice with PBS and cultured with fresh medium (recovery studies) or immediately fixed in ice-cold 70% ethanol for 1 hr, stained with propidium iodide/RNase (Trevigen Inc., Gaithersburg, MD) and washed with PBS before analyzed by flow cytometry. The distribution of cells in different phases of the cell cycle was determined with the Multicycle module of the FCS Express V4.0 software.

### Numerical Solution of the PBE Model

Numerical solutions of the stem cell PBE models described in this study were obtained via MC simulations considering the interruption of quiescence in the population by the division of a cell. A similar methodology has been applied to other systems of microbial populations, coalescing droplets and chemical reactions [32], [33], [34]. The time 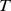 between successive divisions of any two cells (i.e. interval of quiescence between 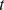 and 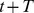) is a random variable with a cumulative distribution function 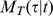 defined as the probability that time 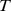 will be less or equal to 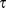 conditional on the state of the stem cell population at instance *t* [35]. If the probability for 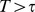 conditional on the state of the population at time *t* is 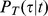, then.
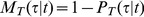
(1)


The probability 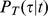 is expressed as
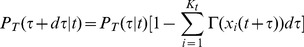
(2)where 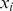 is the state vector for the i^th^ cell in the population of 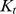 stem cells. The second term on the right-hand side represents the probability that division will not interrupt quiescence during the interval 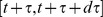. Division of Eq. 2 by 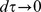 and the initial condition 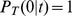 (i.e. the quiescence time is always >0) yield
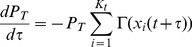
(3)and thus,



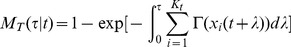
(4)The quiescence interval T can be calculated from the above equation by setting the distribution 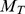 equal to a random number (*ran1*) from a uniform distribution, i.e.
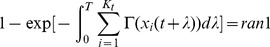
(5)


When only the hESC size was considered in the state vector of the PBE (see Eq. 7), the above expression was coupled to the rate of growth 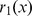 (shown in Eq. 8) which was calculated with the explicit Euler forward difference method for each cell over the quiescence interval and the state vector 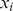 was updated. With the inclusion of the NANOG level in the state vector (Eq. 12), the NANOG content for each cell during the interval 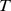 was updated by solving the set of 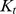 stochastic differential equations (Eq. 13) by the Euler-Maruyama algorithm [36]. Once the time 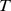 to the next division was calculated (by the Newton-Raphson method), the j^th^ cell undergoing division was identified by generating a second random number (*ran2*) from a uniform distribution to calculate the probability.
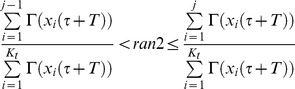
(6)


The cell sizes and NANOG content of the two daughter cells were determined by selecting two more random numbers (*ran3* and *ran4*) from a uniform distribution according to the partition functions 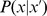 and 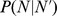 (Eq. 9 and 15), respectively. For the cell size-based PBE, only *ran3* was selected. The initial population size 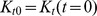 was set to 5,000 hESCs. During simulation, the number of cells increased to an upper limit 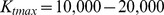 cells as stated (constant volume MC [37]) by replacing the dividing mother cell with its daughter cells in each step. These values for 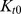 and 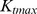 provided adequate resolution for comparison of the simulation results with our experiments. Once 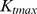 was reached, the algorithm became a constant (cell) number MC [37]. For this purpose, the mother stem cell was replaced by the first daughter cell while another cell from the population was randomly selected and swapped with the second daughter cell. The simulation was terminated when the time reached a predetermined limit (e.g. t_total_ = 16 hr). A description of the algorithm is also shown in Figure S3. Codes were written in FORTRAN and post-processing was performed in MATLAB (Mathworks, Natick, MA).

### Statistical Analysis

Statistical analysis including ANOVA and the post-hoc Tukey test was performed using Minitab (Minitab Inc., State College, PA). P values less than 0.05 were considered as significant. The Pearson’s product-moment correlation coefficient was used to calculate the correlation Corr(x,N) between the size (x) and NANOG (N) in distributions obtained by flow cytometry or PBE model simulations.

## Results

### Size-based Stem Cell PBE Model

Initially the PBE model below was constructed for self-renewing hESCs considering only their size x in the state vector.
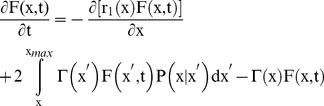
(7)


F(x,t) is the probability density function where F(x,t)dx represents the number of hESCs per unit culture volume with size between x and x+dx at time t. The size x corresponds either to the cell volume or mass since the buoyant density of cells does not vary throughout the cell cycle [38], [39]. The first term on the right-hand side describes the disappearance of cells with size x due to their growth with a rate r_1_(x). Single cell size increases exponentially as was recently shown in an elegant study by Tzur et al. [40], i.e.
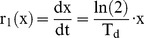
(8)with T_d_ being the hESC doubling time.

The second right-hand side term in the PBE corresponds to the division of a mother cell with size x’ to two newborn cells. Since the partitioning of the content of the mother cell to its daughter cells is random, a partition probability density function 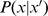 is introduced. This function, which represents the probability that a mother cell x’ will produce two daughter cells with sizes x and (x’-x), was taken to be [41].
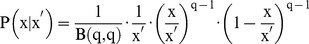
(9)with B(q,q) being a symmetric beta distribution. Obviously, 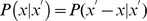 and the total x’ is conserved during mitosis.

The last term in Eq. 7 represents the loss of cells with size x due to their division. The mitotic cell size conforms to a Gaussian distribution G(x) (with mean μ and standard deviation σ) [40] allowing to deduce the following function [41], [42] for the cell division rate.
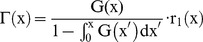
(10)


Formulation of the PBE assumes that all hESCs actively proliferate and cell death is negligible. Cultured hESCs exhibit a short G_1_ phase minimizing the likelihood of their entrance to the G_0_ state [26], [29]. As a result, the vast majority of hESCs remain mitotically active in culture [29]. Moreover, we routinely measure over 90–95% viability of self-renewing hESCs.

**Figure 1:**
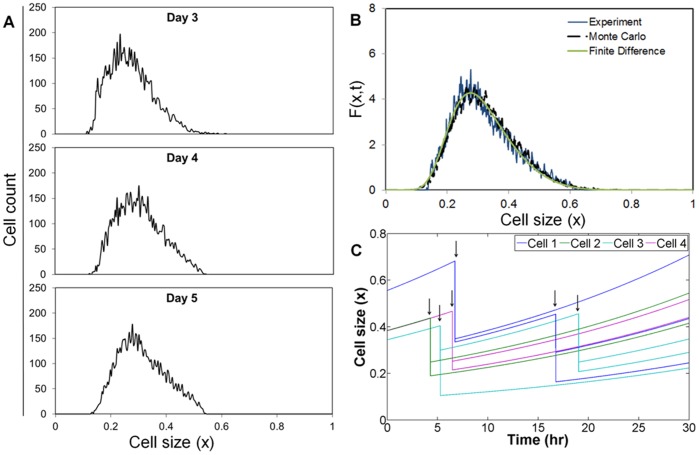
Human ESC size distributions obtained by flow cytometry and simulation. (A) FSC (transformed to normalized cell size) data distributions at day 3, 4 and 5 after plating hESCs on Matrigel-coated dishes. Each distribution is representative of at least three independent experiments. (B) Simulation results are shown of the cell size-based PBE using the MC and finite difference methods. Experimental data obtained by flow cytometry are also shown. Estimation of parameters values from experimental data is described in the text. (C) Model predictions of the size dynamics of four randomly selected hESCs and their daughter cells. Division events are denoted by arrows.

The cell size in the state vector of the PBE was obtained by flow cytometry utilizing the data in the forward light scatter channel (FSC) [43], [44], [45] (Fig. S2). Human ESCs were heterogeneous with respect to size as relevant distributions show (Fig. 1A). The volume of hESCs was defined through the variable 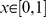 normalized from FSC data. Size-wise the hESCs maintained a time-invariant state and we chose the FSC-based size distribution at day 5 as the initial probability density function (F(x,0)) for our simulations. Parameters for the cell division probability Γ(x) and the partitioning function P(x|x’) were obtained from FSC data (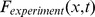) by coupling the solution of the PBE (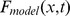; Fig. 1B) to a minimization of the objective function.


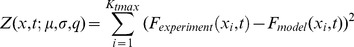
(11)

by the Nelder-Mead algorithm [46]. Data from at least seven experiments were used for parameter estimation (Table 1). It should be noted that the calculated values for μ, σ and q satisfied relevant constraints (0<μ<1, σ>0 and q>0). In addition to the MC method described above, we also implemented a time-explicit finite difference method by discretizing the cell size domain via a hybrid leapfrog/Lax-Friedrichs scheme [41] and imposing regularity conditions at the boundaries [47]. The solutions obtained from both algorithms were in excellent agreement (Fig. 1B). Moreover, the temporal evolution of the size of individual hESCs can be tracked with the model (Fig. 1C). Taken together, the stem cell size-based PBE model illustrates that self-renewing hESCs in culture exhibit a stable size distribution over time as reflected by the FSC data.

**Table 1:** Parameter values calculated based on data from experiments (n = 3–7).

Parameter	Mean ± st. dev.
*μ*	0.463±0.048
*σ*	0.100±0.025
*q*	39.519±2.291
*α* (x10^3^ molecules/hr)	7.87±0.239
*d* (x10^−2^/hr)	7.61±0.591
*δ* (x10^4^ molecules/hr)	1.74±0.0039

### Stem Cell PBE Model: Cell Size and NANOG Expression

Our objective was to investigate the contribution of stochastic partitioning during cell division to the NANOG profile exhibited by hESCs. With the parameters related to hESC size and growth calculated from experimental data, the model was extended to include the intracellular NANOG level:
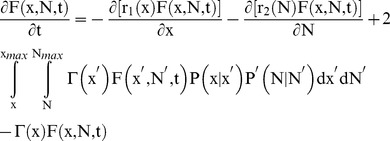
(12)


A schematic of the PBE model is depicted in Fig. S4. Here, F(x,N,t)dxdN represents the number of stem cells per unit volume of culture with size between x and x+dx and NANOG expression between N and N+dN at time t.

NANOG expression dynamics were modeled based on the stochasticity of gene transcription with the noise propagating to the NANOG protein production (see Methods S1 for derivation of r_2_(N)):


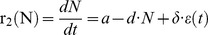
(13)

The stochastic differential equation includes a white (Gaussian) noise term δ^.^ε(t) centered at 0 and with a standard deviation (noise amplitude) δ, which models heuristically the effect of stochastic fluctuations in the *NANOG* expression, e.g. due to transcription and translation [48], [49]. NANOG was assumed not to affect the hESC division potential (Eq. 10), i.e.
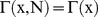
(14)


Stochasticity in NANOG partitioning during division was introduced with the function P(x,N|x’,N’). We assumed that cellular content (size x) and NANOG (N) is divided independently leading to factorization of the partitioning probability density function:
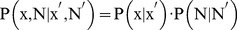
(15)


The same symmetric beta distribution was assumed for P(N|N’) (so that 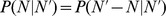) as for cell size partitioning (Eq. 9) with the same parameter q.

Concurrently with the development of the model, we analyzed the expression of NANOG in self-renewing hESCs by flow cytometry. Before proceeding further with our analysis, we examined the adaptation status of cultured hESCs [50]. Human ESC culture adaptation typically results in extensive loss of heterogeneity with differences in morphology and the reduced potential for lineage commitment compared to normal hESCs. For instance, hESCs in our cultures formed colonies with well-defined edges and surrounding fibroblast-like cells were observed (Fig. S5A–B) as expected for normal hESCs and in contrast to cultures of hESC variants [50]. The cells also maintained a normal karyotype (Fig. S5C). Moreover, hESCs subjected to directed differentiation expressed markers of mesoderm, ectoderm and definitive endoderm (Fig. S5D–F). Human ESC-derived definitive endoderm cells were further coaxed to posterior foregut cells expressing PDX1 (Fig. S1). These data provide evidence that our hESCs did not resemble culture-adapted hESCs, which typically represent a population with higher homogeneity.

**Figure 2:**
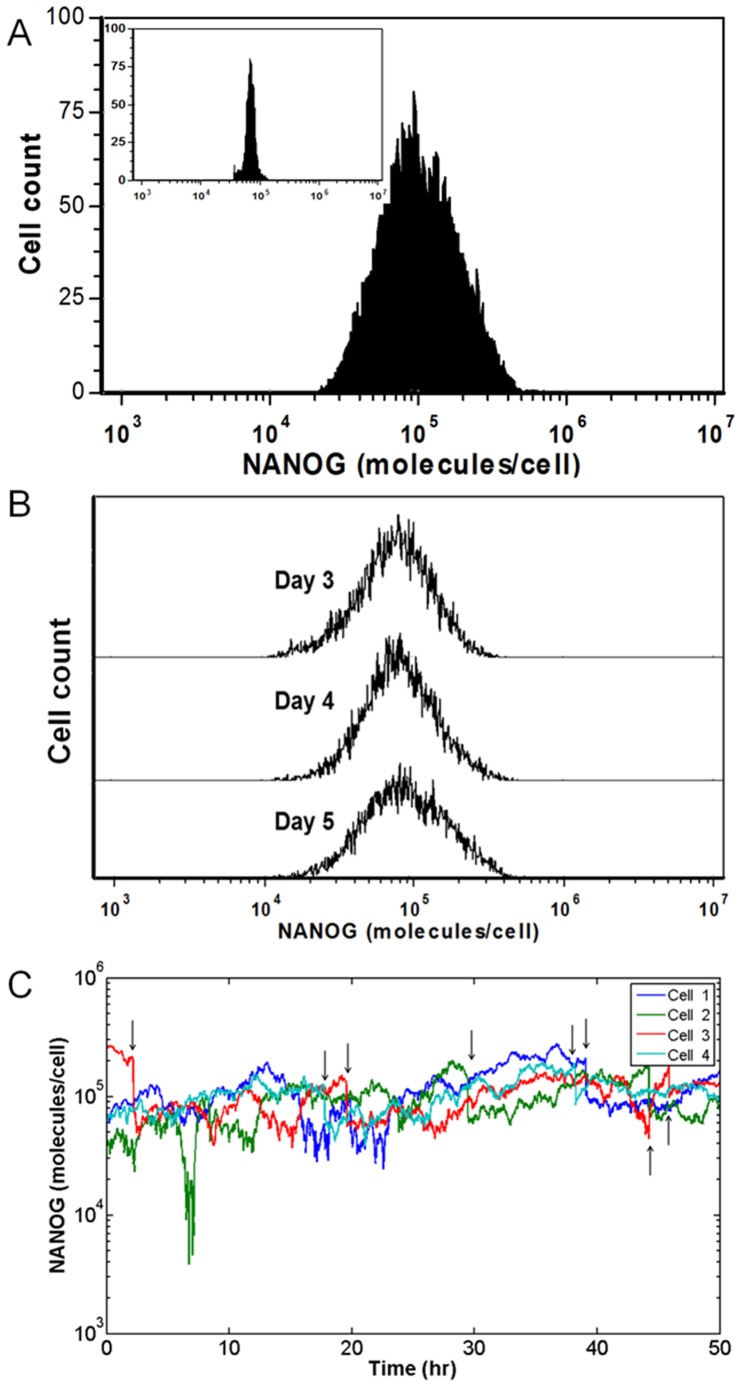
Heterogeneous NANOG expression by hESCs. (A) NANOG expression of hESCs measured by flow cytometry. Arbitrary units (AU) were converted to NANOG molecules per cell with the use of MESF beads (inset). (B) NANOG expression profiles of cultured hESCs at day 3, 4 and 5 after plating. (C) Simulated NANOG dynamics for four randomly chosen cells. Division events are denoted by arrows.

The heterogeneity of NANOG expression among hESCs becomes evident when the dispersion of NANOG profile (Fig. 2A) is compared to that of MESF beads. The distribution of NANOG by cultured hESCs on day 3 exhibited significantly greater dispersion than that of MESF beads as the respective coefficients of variation (CV) were 0.632 and 0.19 (p = 3.14×10^−3^, n = 3). As with hESC size, the NANOG distribution remained fairly constant over time under routine culture conditions (Fig. 2B). The CV of NANOG protein distribution did not change significantly 4 (0.588) or 5 (0.643) days after passaging. Taken together, our data show that self-renewing hESCs maintain a polydispersed and stable distribution of NANOG expression.

**Figure 3:**
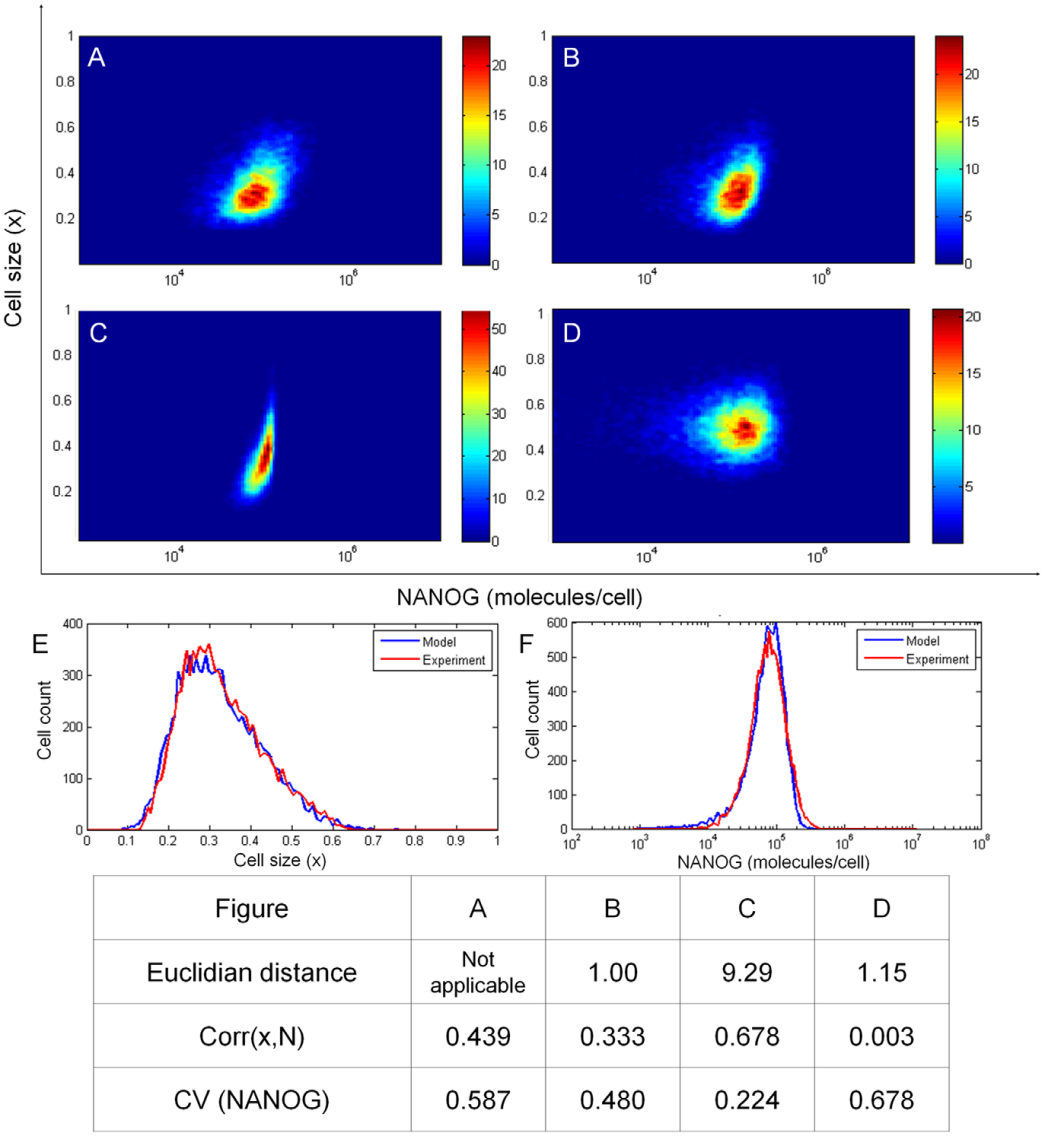
Comparison of PBE simulation results with data from experiments on hESC size and NANOG expression. Data from (A) hESC cultures were compared to those from the model considering (B) both cell division and NANOG expression noise, (C) only cell division, or (D) only NANOG expression noise. In (B)-(D), the model was run for an interval of four doubling times. Color bars indicate cell count values (zero moment of the density function). Plots are based on 10,000 cells. (E–F) Comparison of cell size and NANOG expression distribution between experiment and PBE model with both cell division and NANOG expression distribution considered. Euclidian distance was calculated with respect to the experimental data set. Correlation coefficients for hESC size and NANOG as well as the CV for NANOG distributions are also listed. Cell size was normalized to [0,1].

Flow cytometry data for NANOG were then used to evaluate parameters pertinent to the expression dynamics of NANOG. For this purpose, NANOG data (AU) were converted to actual NANOG molecules/cell utilizing the MESF beads (Fig. S2). The PBE was solved by the MC method based on interval-of-quiescence calculations. During each interval, the NANOG content was updated for each cell by solving the set of *K_t_* stochastic differential equations (r_2_(N)) whereas changes in cell size were calculated according to r_1_(x). Values for α, d and δ (Table 1) in r_2_(N) were calculated from experimental data through minimization of the objective function Z(x,t;a,d,δ) as in Eq. 11. Of note, the model delivers information on the temporal evolution of NANOG in individual cells (Fig. 2C). With these parameter values, simulations yielded results which were in close agreement with the data acquired from experiments (see below Fig. 3).

Collectively our findings show that self-renewing hESCs exhibit stable but heterogeneous patterns of size and NANOG expression (Movie S1) in line with previous reports. The stochastic PBE model developed based on experimental data captures the NANOG heterogeneity of hESCs taking into account the noisy gene expression and stochastic partitioning during mitosis.

### Gene Expression and Cell Division Effects on NANOG Expression

With the PBE framework in place, we set out to investigate the impact of partitioning at cell division and expression noise on the NANOG profile of self-renewing hESCs. Simulations were run in the presence or absence of these two sources of heterogeneity (Fig. 3). The Euclidian distance was calculated as a measure of divergence between simulation and experimental data (Fig. 3A). This was found to be the lowest when both cell division and NANOG expression noise were considered (Fig. 3B). In addition, two scenarios were run: (i) Cells continued to proliferate with deterministic NANOG kinetics (i.e. the noise term in r_2_(N) was set to zero; Fig. 3C). (ii) Cells expressed NANOG stochastically and grew with rate r_1_(x). Once a cell was determined as ready to divide (equivalent to G_2_/M phase), it was placed in a blocked-division pool where cells did not grow further (i.e. r_1_(x) = 0; see Fig. S3). The results from both scenarios diverged from the experimental data even though to a lesser extent when only cell division was blocked (Fig. 3D).

The difference in the above scenarios was also mirrored in the correlation (Corr(x,N)) of x and N. Overall, the hESC size and the level of NANOG should increase concomitantly during the cell cycle leading to a positive correlation under non-differentiating conditions without any implied causal relation between the two variables. Indeed, a correlation of 0.439 was calculated from flow cytometry distributions of x and N and this was close to the value (0.333) calculated from the full PBE model. Correlation values were significantly different when only cell division (0.678) or gene expression (0.003) was considered. These findings demonstrate that the stochastic PBE model is aligned with the behavior of the hESC system observed in our experiments and that both sources influence the NANOG profile in hESCs.

To further explore the relative contributions of stochastic partitioning at cell division and transcriptional noise, we examined the CV of NANOG distributions from the simulations described above. The CV for the flow cytometry data of NANOG expression was 0.578 (n = 3) while full model simulations yielded results with a CV of 0.48. A distribution of NANOG (Fig. 3E–F) with a CV value closer to that of the flow cytometry data was obtained when only NANOG expression noise was taken into account. Nonetheless, there was a pronounced discrepancy between the experimental data and simulation for dividing hESCs without transcriptional stochasticity. In this case the distribution appeared narrower (CV = 0.224). These results indicate that gene expression stochasticity has a more pronounced influence on NANOG heterogeneity than cell division alone. Yet, our findings support the notion that both gene expression noise and cell division are important for the emergence of the observed NANOG variation in hESC populations.

### NANOG Expression Shifts when hESCs are Reversibly Arrested in G_2_/M Phase

Our analysis thus far indicated that the NANOG profile is influenced by both stochastic partitioning at cell division and NANOG expression dynamics. The inclusion of transcriptional noise in the model yields results with a CV closer to that of the data from experiments. However, NANOG partitioning contributes less to its heterogeneity. This finding led us to hypothesize that inhibiting cell division should not affect extensively the heterogeneous profile of NANOG in self-renewing hESCs. To that end, hESCs were growth-arrested in culture by incubation with nocodazole or colcemid as this method has been successfully used for hESC cycle analysis [28], [51]. Our results were compared with the predictions from the stochastic PBE model.

**Figure 4:**
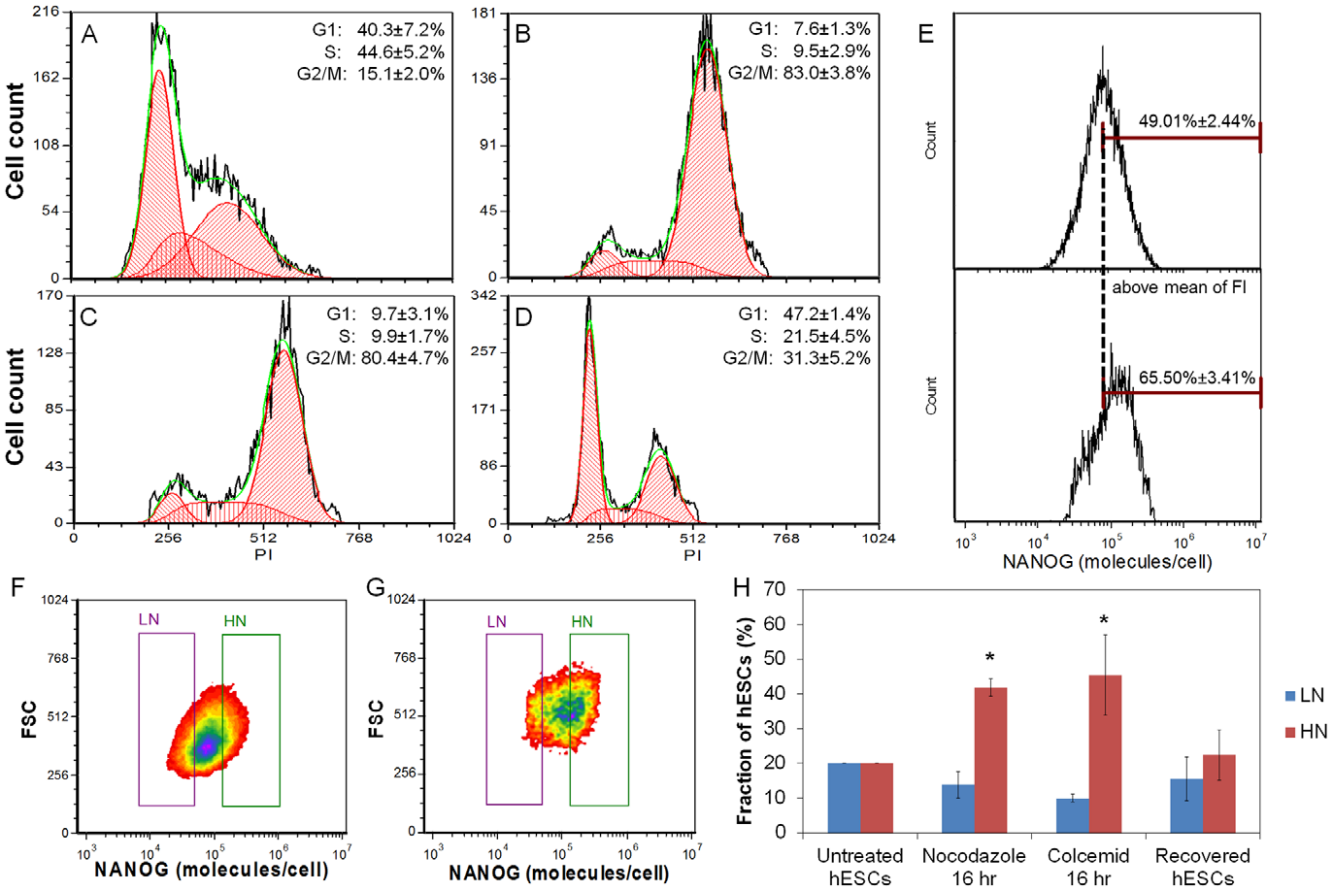
Proliferation arrest of hESCs and NANOG expression distribution. (A–D) Cell cycle analysis performed via flow cytometry on (A) untreated hESCs, and hESCs treated with (B) 200 ng/ml nocodazole or (C) 100 ng/ml colcemid for 16 hrs. (D) Human ESCs after a 2-hr recovery following nocodazole treatment as in (B). Flow cytometry data (black curves) were analyzed using the FCS Express 4.0 software (red, green curves). Results are shown as mean ± standard deviation from at least three independent experiments. (E) Histograms of NANOG expression for (top) untreated and (bottom) nocodazole-treated self-renewing hESCs. The dashed line denotes the mean NANOG fluorescence intensity (MFI) of untreated hESCs. The fractions (%) show the cells with fluorescence intensity above the MFI value. (F) The LN and HN regions were defined by 20% of hESCs with the lowest and highest NANOG expression, respectively. (G) The same gating criteria were applied to Nocodazole-treated NANOG^+^ hESCs; (H) The percentage of LN and HN hESCs under the conditions indicated: (i) Untreated hESCs (normal hESCs), (ii) hESCs treated with 200 ng/ml Nocodazole or (iii) 100 ng/ml colcemid for 16 hr, and (iv) hESCs recovered 24 hr after a 16-hr treatment with nocodazole. Error bars are calculated from at least three independent experiments (*p<0.001).

In untreated cultures 40.3±7.2% and 44.6±5.2% of the hESCs were in the G_1_ and S phases respectively, whereas only 15.1±2% was in the G_2_/M phase (Fig. 4A). Treatment with 200 ng/ml nocodazole resulted in a significant reduction in the number of hESCs in the G_1_ (7.6±1.3%) and S phases (9.5±2.9%) with a concomitant increase of the G_2_/M phase hESCs (83%±3.8%; Fig. 4B). A similar shift was noted when hESCs were treated with colcemid (Fig. 4C) suggesting that the results were not specific to the particular drug used but due to the mitotic arrest of the cells. Furthermore, this shift was reversible since withdrawal of nocodazole reduced the G_2_/M phase cell fraction and increased the cells in the G_1_ and S phases restoring the pre-treatment distribution of cells in the cycle (Fig. 4D). Thus, nocodazole blocks division in a reversible manner arresting hESCs in the G_2_/M phase in line with previous reports [28], [29].

Flow cytometry analysis of nocodazole-treated hESCs showed a 39% increase in the mean fluorescence intensity for NANOG levels, i.e. to 7.47×10^4^ from 5.37×10^4^ (p = 0.0038) NANOG molecules/cell for untreated hESCs (Fig. 4E). Interestingly enough, the percentage of nocodazole-treated NANOG^+^ hESCs with NANOG molecules/cell greater than the corresponding mean value of the untreated hESC populations was 68% (p<0.05). Yet, NANOG heterogeneity measured by CV for the untreated (CV = 0.646) and growth-arrested cells (CV = 0.67) was unchanged.

To better demonstrate the effect of division block on NANOG distribution, we analyzed the NANOG^+^ hESC population by defining NANOG^low^ (LN) and NANOG^high^ (HN) regions in the density plot of NANOG vs. FSC for untreated hESCs (Fig. 4F). Each of the LN and HN regions included 20% of the cells with the lowest and highest NANOG expression levels, respectively. The same regions were overlaid on the NANOG (vs. FSC) distribution of nocodazole- or colcemid-treated hESCs (Fig. 4G) and compared to those for untreated hESCs (Fig. 4H). The number of hESCs in the HN region increased significantly from 20% (untreated cells) to 41.77±2.02% (nocodazole-treated; p = 1.17×10^−3^) and 45.43±9.43% (colcemid-treated; p = 0.021). This was accompanied by a decrease in the fraction of hESCs in the LN region. The observed fractions remained constant after a 12, 16 or 20 hr treatment of hESCs (Fig. S6). Interestingly enough, the distribution of NANOG expression was restored to its initial state 24 hr after withdrawal of nocodazole (Fig. 4H). The data collectively show that after blocking division of hESCs the average NANOG expression increased but with no significant change in its heterogeneity.

**Figure 5:**
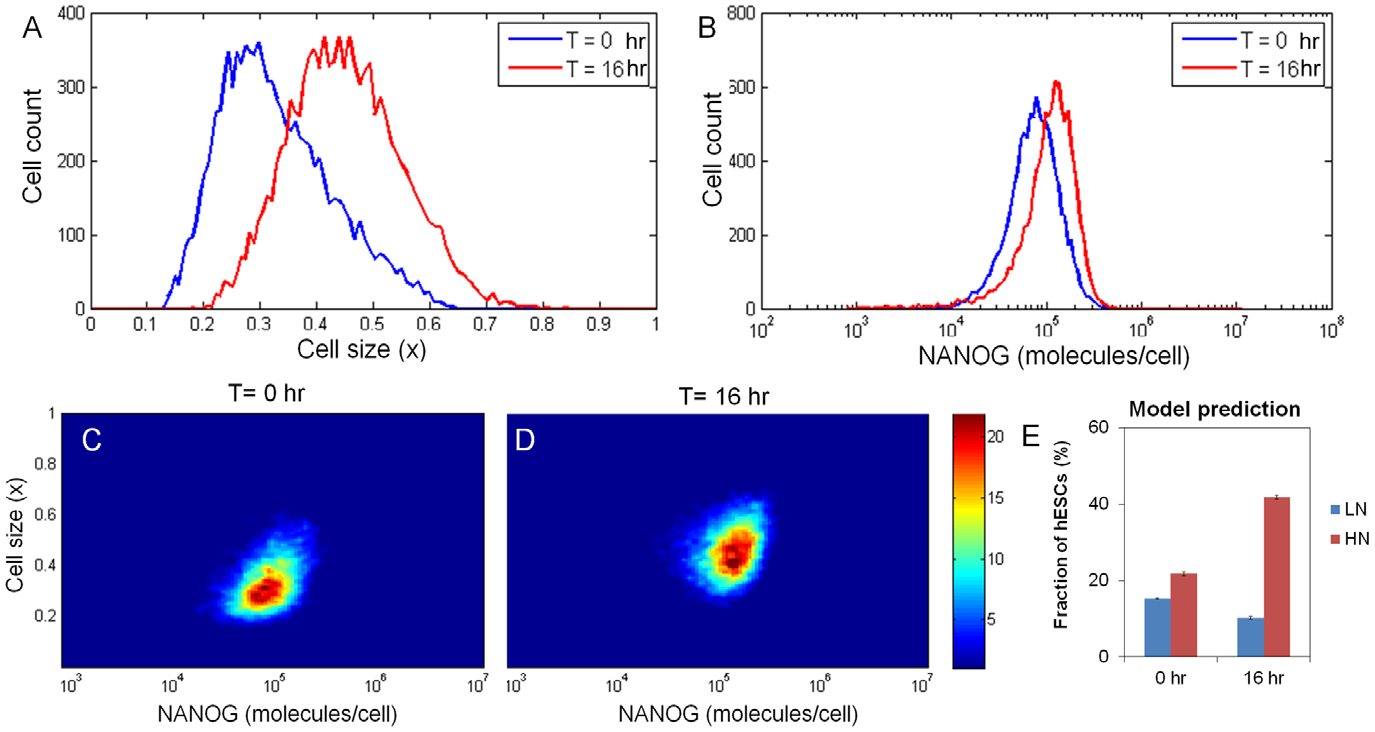
Cell size and NANOG profiles from simulation of hESCs with blocked division. (A) The cell size and (B) NANOG expression are shown for hESCs at t = 0 (blue curves) and after 16 hr (red curves) according to the PBE model with blocked division. Corresponding density plots for (C) size and (D) NANOG are also shown. The density function scale is represented by the color bar. Plots were generated a total of 10,000 hESCs. (E) Model prediction of the fraction of hESCs in LN and HN regions right before and after 16 hr of blocking cell division. Regions are defined as in Figure 4 and results are shown as mean ± std.dev. from three simulations with different random number generator seeds.

Simulating the block in cell division with the PBE model (second scenario in *Gene expression and cell division effects on NANOG expression*), both the hESC size and NANOG expression levels shifted to higher values (Figs. 5A–B) matching the experimental findings. When cell division was blocked, its effect was eliminated, leading to the shift of NANOG distribution at population level as shown in Figures 5C–D (and Movie S2). This is also illustrated in Figure 5E where a significant increase of hESCs in the HN region and a decrease in the LN region were predicted by the model. Nevertheless, the CV for NANOG data generated by the model was 0.544 vs. 0.378 for hESC populations simulated assuming growth arrest. Thus, NANOG heterogeneity is reduced in simulations with the elimination of stochastic partitioning whereas this was not evident from our experiments.

### Cell Division as a Source of NANOG Expression Heterogeneity

The experiments and model simulations revealed that the two processes considered here −partitioning during cell division and gene expression noise− impact the NANOG profile in hESCs. Although transcriptional stochasticity has a significant effect on NANOG expression [21], [22], we discovered that blocking cell division causes a shift in the average NANOG level in hESCs. When cell growth arrest was simulated with the PBE model, the dispersion in NANOG among individual hESCs decreased although this was not apparent from corresponding experiments.

**Figure 6:**
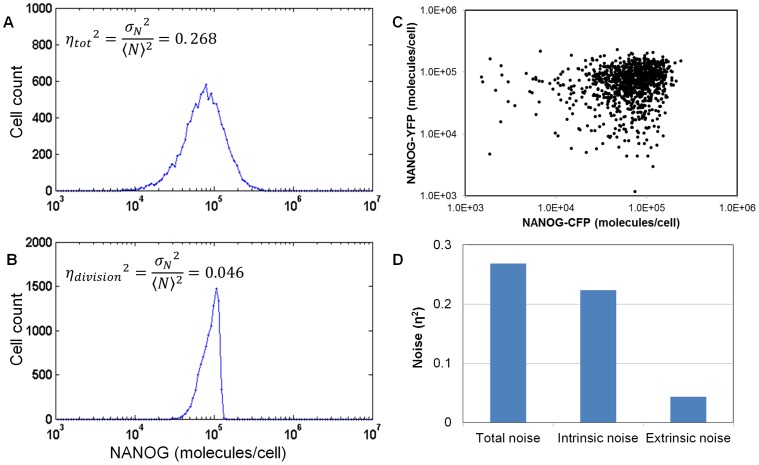
Quantitation of noise in NANOG expressed by hESCs. Total noise in NANOG expression profile (A) obtained by flow cytometry analysis of cultured hESCs and (B) as predicted by the model with gene expression noise set to zero. (C) Scatter plot from simulation of a dual-reporter assay with 10,000 cells expressing NANOG-CFP and NANOG-YFP. Both stochastic partitioning and gene expression were taken into account. (D) Quantification of intrinsic and extrinsic noise components (Eq. 17–19).

Therefore, the model was utilized to quantify the relative contribution of cell partitioning to the observed variability of NANOG in hESCs. To that end, NANOG heterogeneity was represented by the ratio of the variance of the NANOG distribution to the square of its mean (Eq. 16) according to definitions of noise in biological systems [52]. A total noise (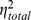) of 0.268 (Fig. 6A) was calculated based on flow cytometry data. Human ESC populations were simulated without transcriptional noise yielding NANOG distributions with a total noise (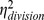) of 0.046 (Fig. 6B). This is 17% of the value of noise obtained from experiments with all sources of heterogeneity present. This result demonstrates that the effect of cell division on cell-to-cell variation is considerable.


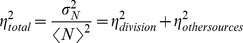
(16)

Similar calculations of noise components have been reported in bacteria [53] and yeast cells [54]. In these systems, single-gene dual reporter experiments are set up to quantify the contributions of intrinsic and extrinsic noise sources on population heterogeneity. Cells are engineered to express two reporter genes (e.g. CFP and YFP) from identical promoters. The difference in the fluorescence of the two reporters is attributed to the inherent stochasticity of transcription and translation (intrinsic noise) [53]. Fluctuations in other cellular components linked to these processes lead indirectly to the same variation of both reporters within individual cells and are considered extrinsic noise.

Engineering hESCs to express NANOG proteins fused to reporter proteins such as CFP and YFP would entail considerable technical hurdles. Yet, such ‘virtual’ experiment can be carried out with the PBE model described here (Fig. 6C). To that end, the PBE state vector comprised three variables, i.e. cell size, and two NANOG variants such as NANOG-CFP and NANOG-YFP ([x Nc Ny]). Its solution was achieved using the same MC algorithm as in the case of the 2D (t,x) and 3D (t,x,N) PBE models. Each reporter partitioned independently after cell division into the two newborn cells according to same partitioning function P(N|N’) used for NANOG in the 3D PBE model. The total noise and its contributions from intrinsic and extrinsic sources was calculated as [52]:
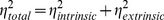
(17)

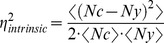
(18)

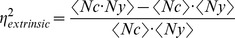
(19)where 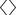 is the expected value of a variable (e.g. the mean of NANOG-YFP intensities is 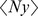). For this purpose, the expected values were calculated from the respective probability density functions of the two NANOG variants based on the solution of the stochastic PBE (Fig. 6A). The intrinsic noise contributing to the heterogeneity of NANOG was quantified to be 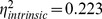 while the contribution of extrinsic noise was 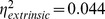 (Fig. 6D). The extrinsic noise was about 17% of total noise (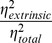) i.e. the same as the result obtained above for the calculation of 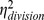. Most notably, the full model was employed for the calculation of intrinsic and extrinsic noise components without setting the gene expression noise to zero unlike in the computation of 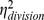.

Overall, the stochastic PBE model presented here allows the calculation of the relative contribution of stochastic partitioning accompanying cell division to the heterogeneous expression of NANOG in populations of self-renewing hESCs. This is a first attempt to estimate intrinsic and extrinsic noise contributions to the profile of NANOG in hESCs. The results show that partitioning is an appreciable −although not the predominant− source of NANOG heterogeneity.

## Discussion

The present study was undertaken to quantify the effect of partitioning during cell division in conjunction with gene expression noise on the NANOG profile of populations of self-renewing hESCs. Although multiple reports have focused on gene expression dynamics, the role of cell division in the observed heterogeneity of stem cell populations has not been studied. To that end, a stochastic PBE model was developed for the first time to describe populations of hESCs based on proliferation and expression of the pluripotency marker NANOG. In agreement with our experiments, the model predicted that the average expression of NANOG increases when mitosis (and thus partitioning) is blocked. Moreover, hESC division-associated partitioning contributes 17% of the total noise exhibited by the NANOG profile. These findings may ultimately provide insights into mechanisms of NANOG regulation and the observed heterogeneity in pluripotent stem cell populations. The computational framework discussed here will aid in the development of strategies for the efficient generation of differentiated progeny for therapeutic applications.

The role of stochastic gene expression in the heterogeneity of isogenic bacteria and yeast cell populations is well-documented [48], [55], [56], [57], [58], [59] but its effects on the non-genetic diversity of stem cell ensembles are less well understood. A model has been described for Nanog in a genetic circuit with Oct4 as its repressor and featuring transcriptional noise as a source of population variability [21]. Bimodal distributions of Nanog (Nanog^low^, Nanog^high^) have been predicted for mESCs under the assumptions of particular gene network kinetics and ergodicity for the cell population. A similar model of Nanog and Oct4-Sox2 heterodimers has yielded comparable Nanog profiles [22]. Mouse hematopoietic progenitor cells reportedly exhibit metastable states linked to noise operating on the transcription of the Sca-1 (*Ly6a*) gene [56].

Nevertheless, a significant gap remains in our knowledge regarding the role on population heterogeneity of cell division and the concomitant random partitioning of cellular material among daughter cells. In the aforementioned study [21], sorted Nanog^low^ mESCs reconstitute the Nanog profile of the original mESC population in 11 days. This period is well beyond the time scale of gene expression processes making cell doubling time pertinent to the heterogeneity exhibited by the population. Splitting of cellular material at division unavoidably alters the levels of various molecules including pluripotency regulators and/or transcription factors. Indeed, mitotically-arrested hESCs have higher levels of NANOG than hESCs with an intact cycle. Consequently, the amount of NANOG in each stem cell is determined not only by the Nanog mRNA/protein production and decay rates but also by Nanog dilution due to cell division. Given that protein dilution rate is decided by the cell cycle time, division most likely affects the levels of other transcription factors as well. Accordingly, Eden et al. [60] reported that division-induced dilution is a major determinant of protein levels in human lung cancer cells, which exhibit similar cycle kinetics with hESCs, and its effect may even surpass those of protein degradation.

Aided by our model, we calculated the noise related to stochastic partitioning in two ways. First, we considered the PBE solution taking gene expression to be deterministic. This led to a division-related noise that was 17% of the total noise in the NANOG profile of self-renewing hESCs. Second, a ‘virtual’ dual reporter experiment was performed similar to those reported for bacteria and yeast cells [53], [54] for the analysis of noise in terms of its broadly defined intrinsic and extrinsic components. The ratio 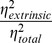 was also found to be 17% considering the distributions of the NANOG variants returned by the full model without eliminating either source of heterogeneity. The agreement between 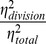 and 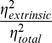 may be in part due to the fact that we only considered stochastic partitioning during mitosis (extrinsic) and gene expression (intrinsic) noise in our model. Yet, in an actual dual reporter experiment will be more challenging to deconvolve the various noise components as other sources of heterogeneity are likely to come into play. For instance, the levels of RNA polymerases and other transcription factors, which are themselves gene products, act on the expression of NANOG and may contribute to its fluctuation. Nevertheless, the 17% of total noise attributed to partitioning in our study is in line with a recent report [61] in which the combined noise due to cell division and protein degradation ranges between 33% to 75% of the total noise of the population. To our knowledge, this is first time the effect of stochastic partitioning on NANOG expression is quantified.

Since partitioning influences the amount of stemness factors in hESCs, it is also expected to affect cell fate determination. A less than two-fold increase in Oct4 leads to differentiation of ESCs to primitive endoderm and mesoderm whereas its repression induces trophoectoderm formation [62]. Reduction in Nanog also causes hESCs to exit self-renewal whereas its overexpression suppresses spontaneous hESC differentiation [15], [19], [20] with accelerated G_1_-to-S phase transition by targeting *CDK6* and *CDC25A* [30]. In fact, the G_1_ phase represents a window of increased sensitivity for hESCs exposed to differentiation signals [63]. To that end, the subpopulation of cells prone to differentiate in the presence of suitable exogenous stimuli can be identified by incorporating an appropriate threshold [18] of NANOG expression in the PBE, which provides the temporal evolution of NANOG profile for each cell in the population.

As already mentioned, cultured hESCs were treated with nocodazole or colcemid to arrest their proliferation, thereby minimizing the effect of partitioning at division on population heterogeneity. Nocodazole treatment has been successfully used for hESC cycle and gene (including Nanog) expression analyses [28], [30], [51], [64]. Consistent with these reports, we observed that nocodazole reversibly arrested the proliferation of hESCs and did not cause differentiation or increase death substantially. However, a recent report [65] indicated the irreversible decrease in NANOG expression with concomitant increase in apoptotic hESCs after a 24-hr exposure to nocodazole. The differences between the findings in this report and those in ours (and in the above-mentioned studies) are challenging to explain but may be attributed to variations in the methods for culturing hESCs. It should be noted that the fraction of NANOG^+^ cells from untreated cultures was significantly lower (up to 60%) than in our experiments (over 80%; Fig. S1).

Similar to our observation of increased NANOG after growth arrest of hESCs, Wang et al. [66] reported that mESCs treated with olomoucine II (an inhibitor of cyclin dependent kinases) shift their cycle profile to the G_2_/M phase with a concomitant increase in the protein levels of Nanog and Oct4. Obviously, differences in the regulation of Nanog and Oct4 between hESCs and mESCs cannot be ruled out but blocking division leads to increased Nanog levels and this may be extended to Oct4 as well. Although we have not carried out a detailed analysis of OCT4 expression in our hESC populations, the model presented here can be expanded in a straightforward manner to encompass more pluripotency markers (e.g. OCT4, SOX2). Additional layers of regulation (e.g. gene regulatory networks) specific to each marker can be incorporated in the PBE model (e.g., through rate functions such as r_2_).

When analyzed by flow cytometry, self-renewing hESCs reproducibly exhibited a NANOG profile with a single peak whereas a bimodal Nanog protein distribution was recently reported for mESCs [21]. Whether Nanog exhibits a similar bimodal distribution in hESCs as in mESCs is still unclear. A graded Nanog distribution was observed in hESC lines with GFP knocked in the *NANOG* locus [67]. Discrepancies in measurements of Nanog expression in these lines based on the actual NANOG protein or the GFP fluorescence could arise from differences in the stability of the respective mRNA and/or proteins. Moreover, the NANOG(GFP)^low^ cells most likely have exited self-renewal as suggested by their upregulated expression of differentiation markers. In our experiments, hESCs with potentially very low or undetectable Nanog content may have overlapped with the isotype controls. These were thus marked as NANOG^−^ and were excluded from our analysis of self-renewing hESCs.

Probing the profile of NANOG in hESCs by flow cytometry provides statistical distributions of cell properties (e.g., antigen presentation) with single cell resolution. As such, this technique is well-suited to capture the marker expression variability within clonal hESC populations. We found that self-renewing hESCs maintained time-invariant distributions of NANOG and size (FSC). Even when cells were mitotically arrested, shifts in population properties were manifested within a cell doubling period. Hence, examination of population snapshots by flow cytometry provided sufficient information for the construction of the PBE model considered here [68]. Moreover, potential artifacts stemming from the preparation of cells (e.g. fixation, antibody staining) were minimized with the inclusion of proper isotype controls and standardized beads. Nevertheless, future studies are planned with hESC populations subjected to differentiation, which will affect global gene expression and cell cycle dynamics and their contributions to population heterogeneity. For these studies, time-lapse microscopy/microfluidics [69] for tracking single cells will complement flow cytometry by delivering kinetic information during hESC commitment. This information will be essential for expanding the PBE model to include complex regulatory networks with gene partners of Nanog or other pluripotency factors. Single-cell microscopy data will also facilitate distinguishing between fast stochastic fluctuations in gene expression (as we have assumed in this study for Nanog based on previous reports [21], [23]) and asynchronous deterministic oscillations [22].

Cell PBE models are inherently multiscale as they depict processes with different kinetics (e.g. gene transcription and cell mitosis) within diverse system boundaries (e.g. intracellular, multicellular) to convey information for individual cells and their ensembles. Such models afford great latitude for formulating and testing hypotheses as our adaptation of the PBE to *in silico* dual reporter assays demonstrates. These experiments are challenging to perform with cultured hESCs. In principle, engineering of hESCs with each gene (e.g. *NANOG*) allele fused with a distinct reporter gene is significantly more complex than the genetic manipulation of yeast cells or bacteria and there are no reports of such hESC lines to date. Moreover, there is no guarantee that the activity of the NANOG protein resulting from the translation of the fusion genes will be the same as that of the native gene product.

Nevertheless, a dual reporter line utilizing the *Nanog* locus was recently described for mESCs [70]. The expression of Nanog at various stages in early mouse development (e.g. pre-implantation embryo, late blastocyst) is allelically regulated and similar patterns are exhibited by mESCs in culture under different conditions. Based specifically on the expression of Nanog (but not Oct4) cultured mESCs can be divided into four groups, i.e. one with biallelic expression of the gene, two with monoallelic expression (from each of the two alleles) and one group exhibiting no expression of Nanog. These findings provide compelling evidence that allelic regulation may contribute not only to the observed Nanog heterogeneity and also to Nanog bimodality in mESCs in contrast to proposed mathematical models featuring positive feedback loop processes. It remains to be determined if the same layer of Nanog regulation is active in hESCs. Despite the focus of our study on hESC gene expression noise and mitosis as sources of NANOG heterogeneity, the PBE model could be expanded to encompass separate PBEs for each group of ESCs based on their allelic expression of Nanog (or other pluripotency markers if applicable). Equations will be coupled through functions representing the switching between expression modes (biallelic, monoallelic or no expression). If allelic switching is a feature of Nanog expression in hESCs, then such framework will allow further deconvolution of this kind of regulation from other sources of heterogeneity considered.

The wide applicability of the PBE framework is also evident by its amenability to context-dependent extensions. For instance, a PBE model can be constructed to study the dynamics of stem cell populations in their niche. A mass-structured PBE coupled to material balances for growth factors, extracellular matrix components and nutrients has been presented for mesenchymal stem cell differentiation [71]. Moreover, gene regulation can be incorporated in a PBE (e.g. via r_2_(N) in our study) as mentioned above. Accordingly, this modeling modality can be employed in the context of cell population reprogramming, which is emerging as a highly stochastic process [72]. A PBE framework connecting different moments of the cell mass distribution to operational variables such as the dilution rate and substrate concentration has also been applied to bioreactor cell cultures and their non-linear feedback control [73]. Similar PBE approaches will benefit the development and robust operation of stem cell bioprocesses for the production of therapeutically useful progeny in medically relevant quantities. The multidimensional PBEs required for these applications become exceedingly challenging to handle numerically especially with traditional finite difference and finite element schemes [74], [75]. Such challenges can be tackled with multi-tier algorithms [76], MC and cell ensemble techniques, which have been utilized successfully to simulate six- and thirteen-dimensional population distributions [77], [78], as well as the continuous increase in available computing power. We expect that quantitative approaches complementing experiments will become more commonplace furthering our knowledge in stem cell biology and accelerating the development of enabling stem cell-based technologies.

## Supporting Information

Figure S1 **Specificity of the NANOG antibody utilized in this study.** (A) HEK293 cells, (B) mouse embryonic fibroblasts (mEFs), (C) undifferentiated hESCs and (D) hESC-derived foregut cells were stained with the NANOG antibody (black curve) or isotype control (gray curve). Human ESCs were differentiated toward posterior foregut as described in Methods S1. (E) PDX1 expression of hESC-derived foregut cells was analyzed by flow cytometry after staining with a primary (goat anti-human PDX1/IPF1 antibody, cat. no. AF2419, R&D Systems) and a secondary antibody (donkey anti-goat DyLight 488 antibody, cat. no. ab96931, AbCam). (F–G) Differentiation control cells were treated with the same media but without differentiation factors (see Methods S1). The gray curves in (E), (G) correspond to respective cell samples stained with the secondary antibody only.
(TIF)

Figure S2 (A) Curve of the molecules/MESF bead vs. fluorescence intensity (AU). The curve was used for converting fluorescence intensity units to NANOG molecules/hESC. (B) Comparison of FSC data and cell diameter, area and size distributions [43], [44], [45]. FSC data of hESCs were normalized to the [0,1] and compared to data obtained by image analysis (ImageJ) of hESCs populations with respect to single cell diameter, area and volume (left-side graph). Flow cytometry FSC channel data varies from 0 to 1024 and the normalization is done based on FSC_normalized_ = FSC/1024. A representative data set from day 5 hESCs is shown. On the right-side graph the distribution resulting from the transformation of FSC data (FSC→FSC^3/2^) is shown compared to those of diameter, area and volume of hESC populations.
(TIF)

Figure S3 **Monte Carlo algorithm for numerical solution of the PBE model.** (A) Solution method for the full PBE model and (B) when a block is imposed on cell division.
(TIF)

Figure S4 **Schematic of the PBE considering both cell size and NANOG level in the state vector.**
(TIF)

Figure S5 **Assessment of the adaptation status of cultured hESCs in this study.** (A) H9 hESC cultured on Matrigel-coated dishes with chemically defined medium form colonies with well-defined edges. The arrow indicates cells with fibroblast-like morphology typically found near colonies of normal hESCs. (B) For comparison, hESCs grown on mEFs are also shown. Bars in (A, B): 100 µm. (C) Karyotypic analysis of cultured hESCs. As evidence of their differentiation potential, cells were successfully subjected to differentiation toward (D) definitive endoderm, (E) mesoderm and (F) ectoderm. The expression of characteristic markers for each lineage was assessed by qPCR and immunostaining. In qPCR results, white bars correspond to hESCs, gray bars correspond to hESCs incubated with differentiation medium but no differentiation factors (control) and hatched bars correspond to hESCs subjected to directed differentiation. For *NANOG* and *POU5F1* qPCR: *p<0.05 or **p<0.005 compared to undifferentiated hESCs. For lineage-specific marker qPCR: ^#^p<0.05 compared to control (no differentiation factor) cells. For methods see Methods S1.
(TIF)

Figure S6 **Fractions of hESCs with the lowest (LN) and highest (HN) NANOG content after treatment with 200 ng/ml nocodazole for 12–20 hr.** The LN and HN regions were defined as containing each 20% of the untreated (control) hESCs with the lowest and highest NANOG content, respectively. *p<0.01.
(TIF)

Table S1 **Primers used for qPCR in this study.**
(DOCX)

Methods S1 **Supplemental information.**
(DOCX)

Movie S1 **Changes in the density plots of cell size vs. NANOG for hESCs cultured under normal conditions (self-renewal).**
(WMV)

Movie S2 **Changes in the density plots of cell size vs. NANOG for hESCs cultured under conditions arresting their proliferation.**
(WMV)
