# Supplementary figures and images for: Contribution of Stochastic Partitioning at Human Embryonic Stem Cell Division to NANOG Heterogeneity
Source: PLoS One. 2012 Nov 30;7(11):e50715. doi: 10.1371/journal.pone.0050715 (PMC3511357; doi:10.1371/journal.pone.0050715)

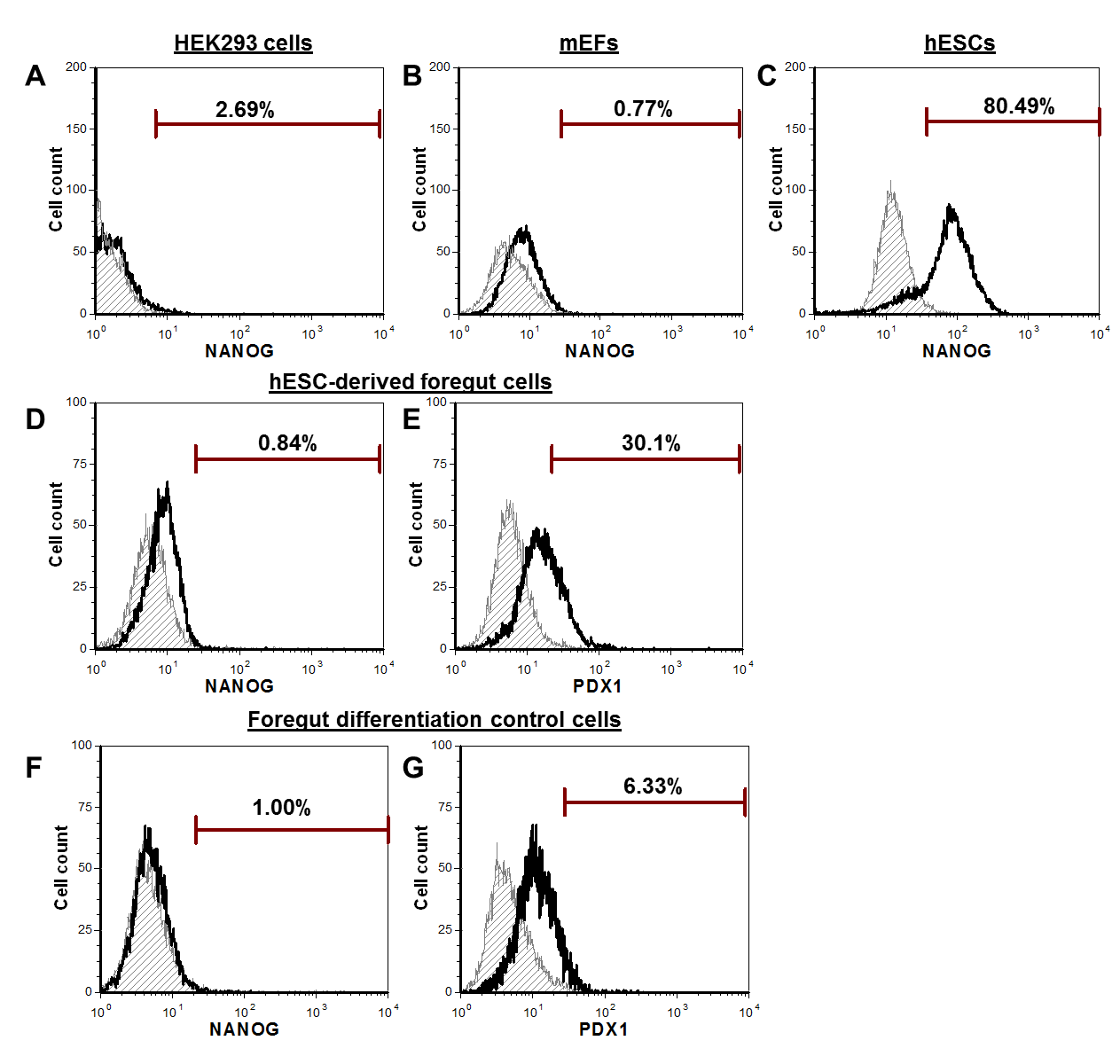

Supplement: Figure S1 — Specificity of the NANOG antibody utilized in this study. (A) HEK293 cells, (B) mouse embryonic fibroblasts (mEFs), (C) undifferentiated hESCs and (D) hESC-derived foregut cells were stained with the NANOG antibody (black curve) or isotype control (gray curve). Human ESCs were differentiated toward posterior foregut as described in Methods S1. (E) PDX1 expression of hESC-derived foregut cells was analyzed by flow cytometry after staining with a primary (goat anti-human PDX1/IPF1 antibody, cat. no. AF2419, R&D Systems) and a secondary antibody (donkey anti-goat DyLight 488 antibody, cat. no. ab96931, AbCam). (F–G) Differentiation control cells were treated with the same media but without differentiation factors (see Methods S1). The gray curves in (E), (G) correspond to respective cell samples stained with the secondary antibody only. (TIF) [file pone.0050715.s001.tif]

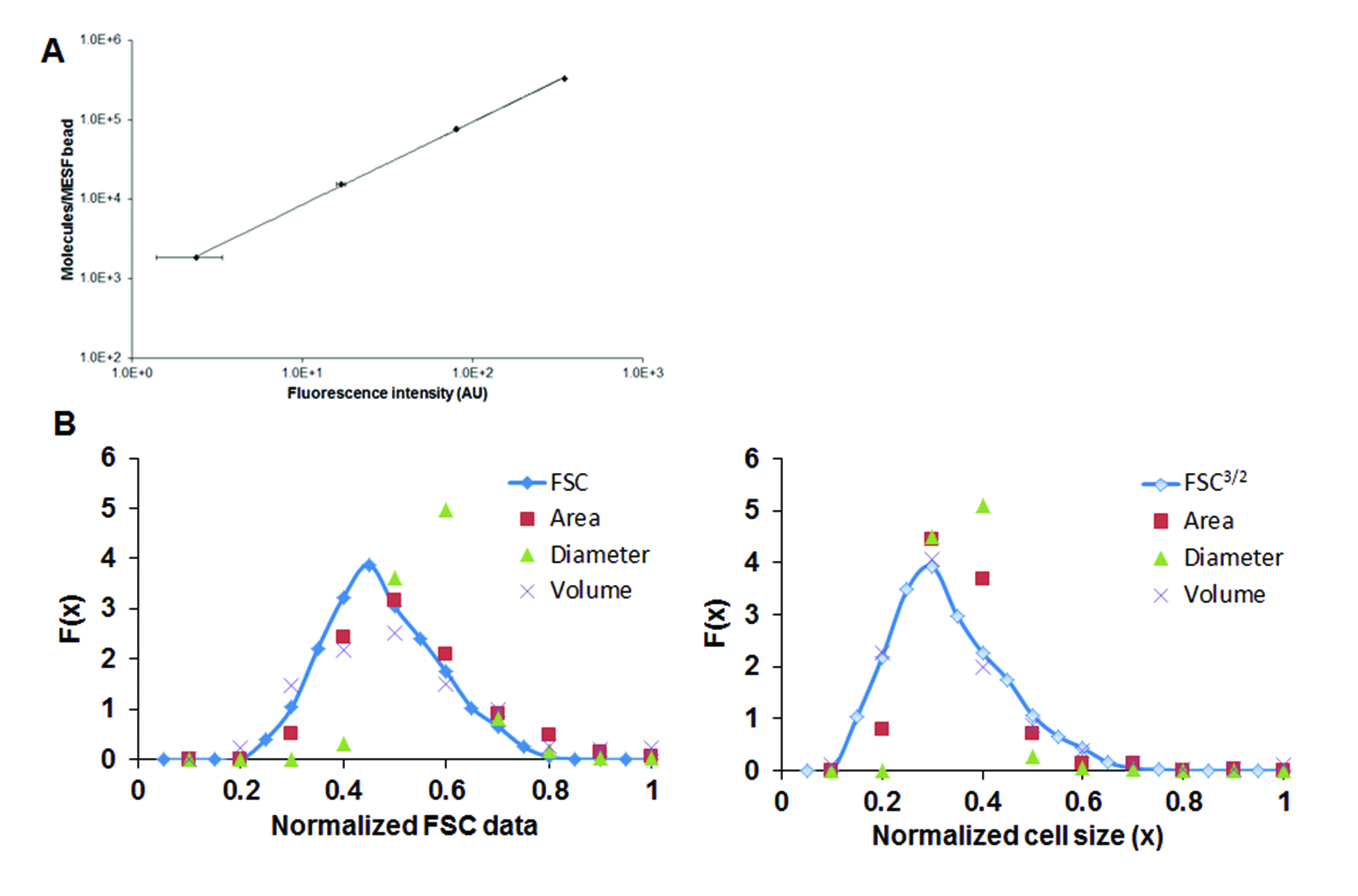

Supplement: Figure S2 — (A) Curve of the molecules/MESF bead vs. fluorescence intensity (AU). The curve was used for converting fluorescence intensity units to NANOG molecules/hESC. (B) Comparison of FSC data and cell diameter, area and size distributions [43], [44], [45]. FSC data of hESCs were normalized to the [0,1] and compared to data obtained by image analysis (ImageJ) of hESCs populations with respect to single cell diameter, area and volume (left-side graph). Flow cytometry FSC channel data varies from 0 to 1024 and the normalization is done based on FSCnormalized = FSC/1024. A representative data set from day 5 hESCs is shown. On the right-side graph the distribution resulting from the transformation of FSC data (FSC→FSC3/2) is shown compared to those of diameter, area and volume of hESC populations. (TIF) [file pone.0050715.s002.tif]

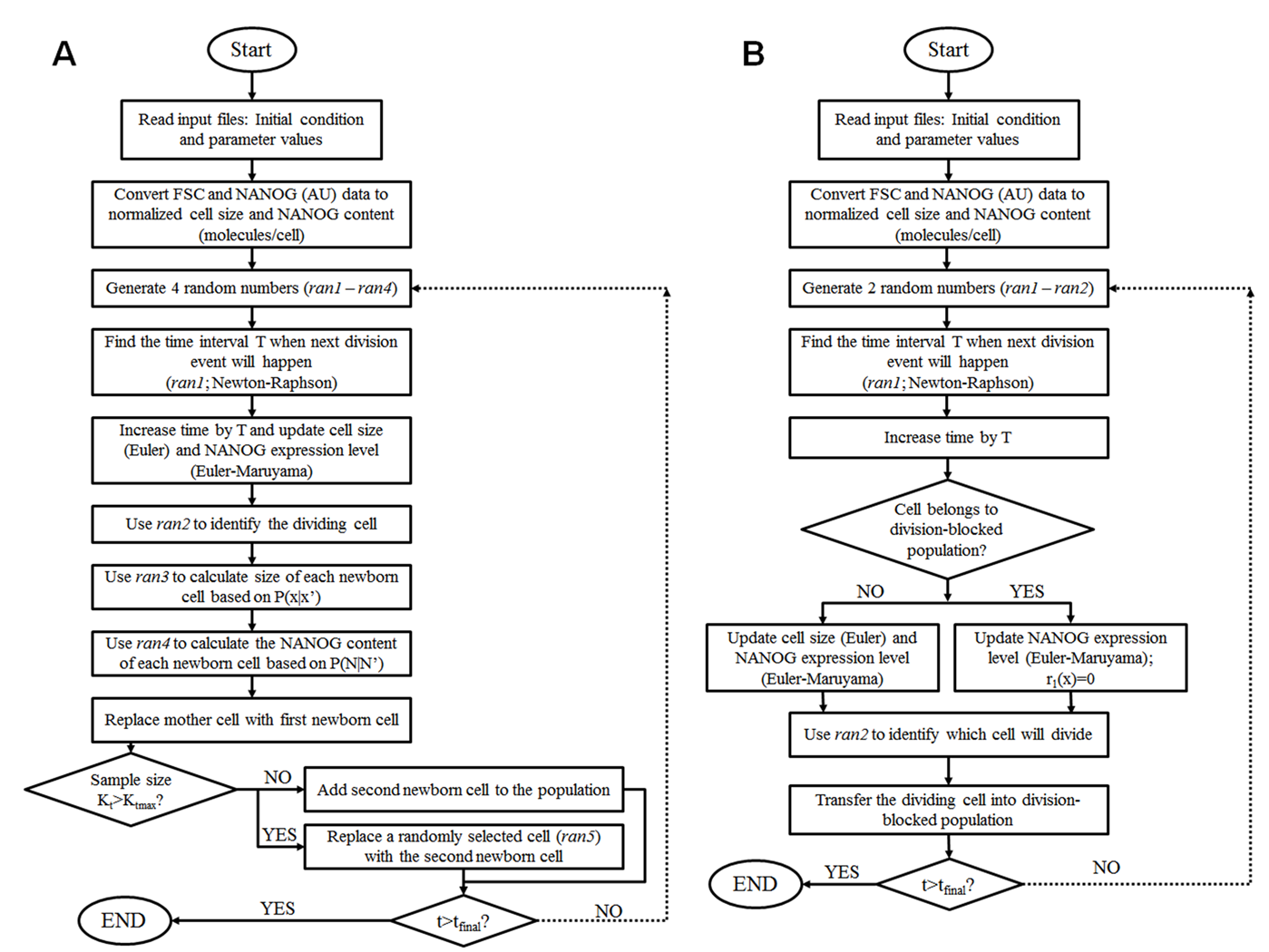

Supplement: Figure S3 — Monte Carlo algorithm for numerical solution of the PBE model. (A) Solution method for the full PBE model and (B) when a block is imposed on cell division. (TIF) [file pone.0050715.s003.tif]

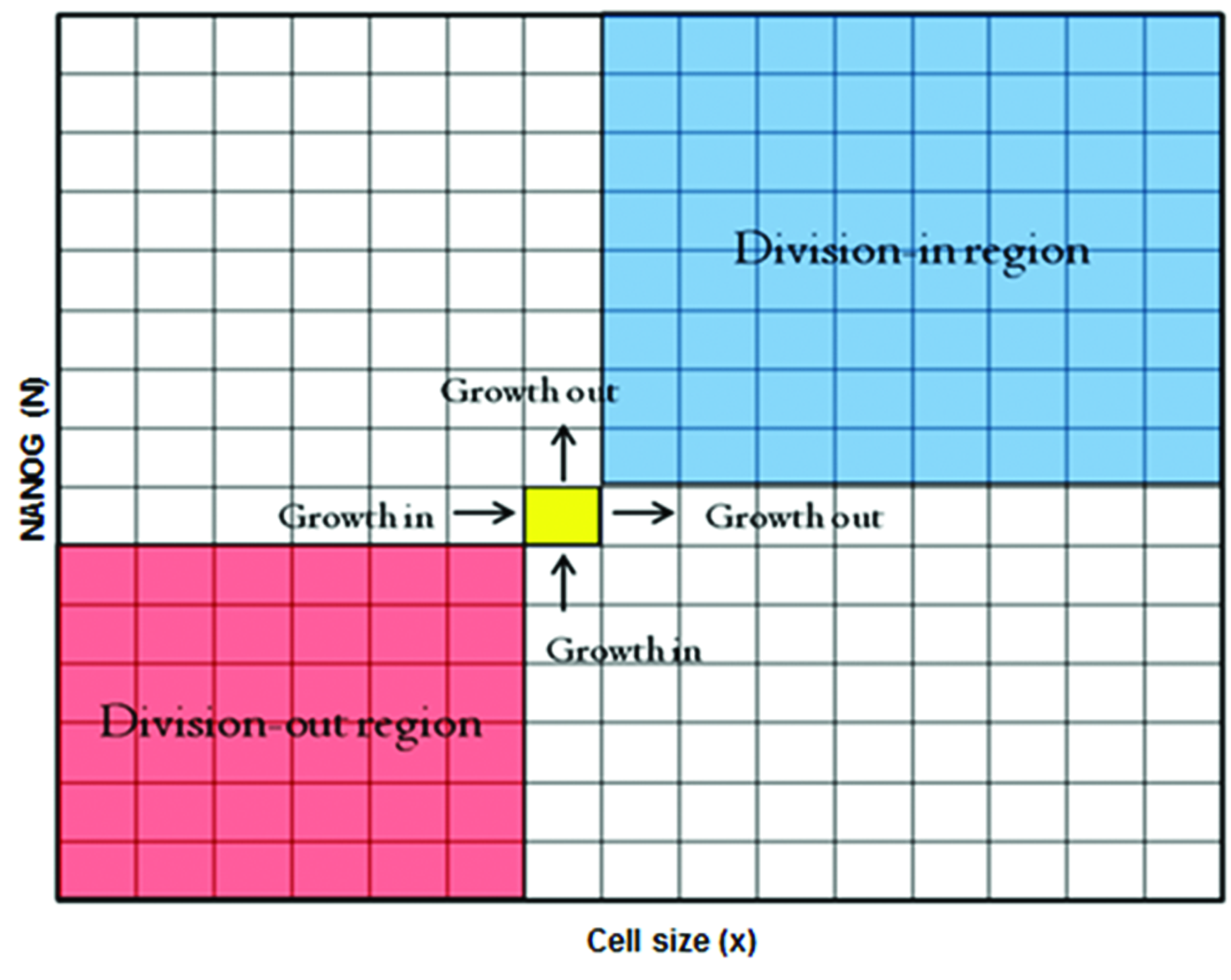

Supplement: Figure S4 — Schematic of the PBE considering both cell size and NANOG level in the state vector. (TIF) [file pone.0050715.s004.tif]

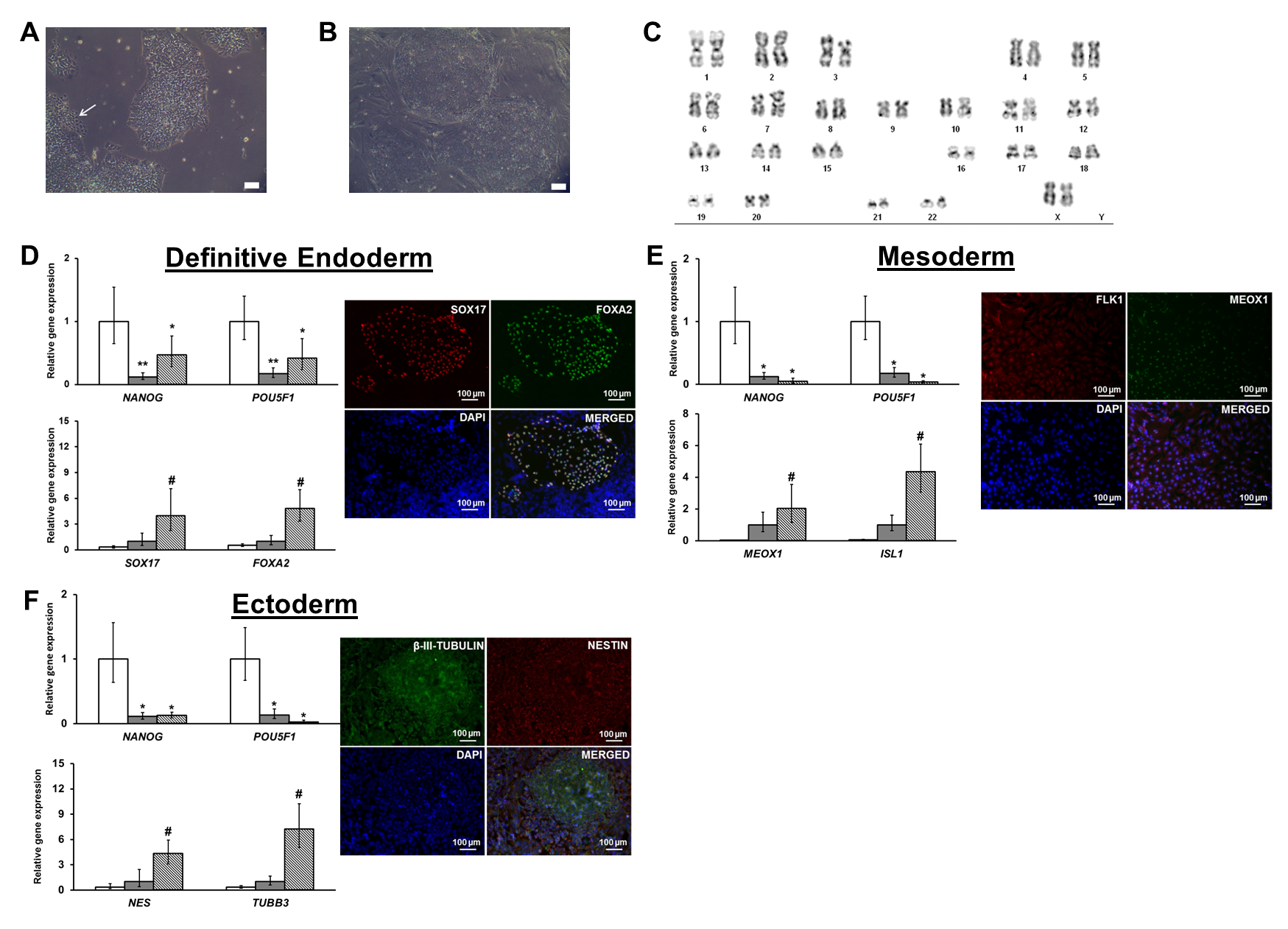

Supplement: Figure S5 — Assessment of the adaptation status of cultured hESCs in this study. (A) H9 hESC cultured on Matrigel-coated dishes with chemically defined medium form colonies with well-defined edges. The arrow indicates cells with fibroblast-like morphology typically found near colonies of normal hESCs. (B) For comparison, hESCs grown on mEFs are also shown. Bars in (A, B): 100 µm. (C) Karyotypic analysis of cultured hESCs. As evidence of their differentiation potential, cells were successfully subjected to differentiation toward (D) definitive endoderm, (E) mesoderm and (F) ectoderm. The expression of characteristic markers for each lineage was assessed by qPCR and immunostaining. In qPCR results, white bars correspond to hESCs, gray bars correspond to hESCs incubated with differentiation medium but no differentiation factors (control) and hatched bars correspond to hESCs subjected to directed differentiation. For NANOG and POU5F1 qPCR: *p<0.05 or **p<0.005 compared to undifferentiated hESCs. For lineage-specific marker qPCR: #p<0.05 compared to control (no differentiation factor) cells. For methods see Methods S1. (TIF) [file pone.0050715.s005.tif]

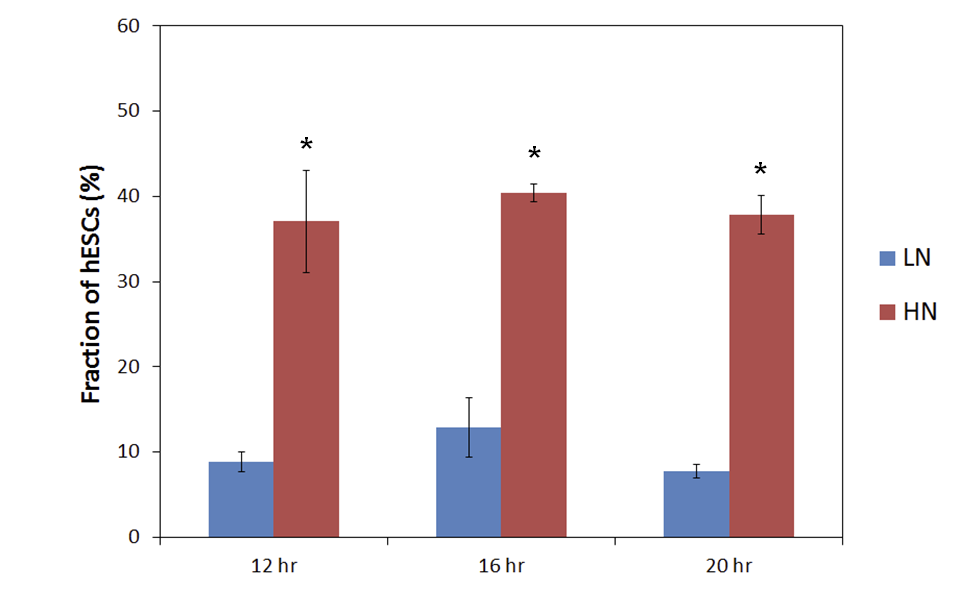

Supplement: Figure S6 — Fractions of hESCs with the lowest (LN) and highest (HN) NANOG content after treatment with 200 ng/ml nocodazole for 12–20 hr. The LN and HN regions were defined as containing each 20% of the untreated (control) hESCs with the lowest and highest NANOG content, respectively. *p<0.01. (TIF) [file pone.0050715.s006.tif]
