# Supplementary material for: Contribution of Stochastic Partitioning at Human Embryonic Stem Cell Division to NANOG Heterogeneity
Source: PLoS One. 2012 Nov 30;7(11):e50715. doi: 10.1371/journal.pone.0050715 (PMC3511357; doi:10.1371/journal.pone.0050715)
Supplement: Table S1 — Primers used for qPCR in this study. (DOCX) [file pone.0050715.s007.docx]

**Table S1.** Primers used in this study. Primers are shown in a 5’-to-3’ orientation.

| Gene name | Forward primer sequence | Reverse primer sequence | Amplicon size (bp) |
| --- | --- | --- | --- |
| β-Actin (*ACTB*) | CTTCCTGGGCATGGAGTCCT | AGGAGCAATGATCTTGATCTTC | 202 |
| *NANOG* | AGATGCCTCACACGGAGACT | ACACAGCTGGGTGGAAGAGA | 194 |
| OCT4 (*POU5F1*) | AAGCTGGAGAAGGAGAAGCTG | AATAGAACCCCCAGGGTGAG | 158 |
| *SOX17* | CTTTCATGGTGTGGGCTAAGG | GTACTTGTAGTTGGGGTGGTCCT | 191 |
| *FOXA2* | GAAGATGGAAGGGCACGA | CACGTACGACGACATGTTCA | 193 |
| *MEOX1* | AGAGTTTGCCCATCATAACTACCT | GCTCAGTCCTTAGTCATTTTTCCTC | 238 |
| *ISL1* | GCGGAGTGTAATCAGTATTTGGA | CACTCGATGTGATACACCTTGG | 184 |
| Nestin (*NES*) | CAGCGTTGGAACAGAGGTTG | GGGAATTGCAGCTCCAGCTT | 289 |
| β-III-tubulin (*TUBB3*) | CTGGAACCCGGAACCATGGA | AGCGAGTGGGTCAGCTGGAA | 215 |
