# Supplementary material for: Contribution of Stochastic Partitioning at Human Embryonic Stem Cell Division to NANOG Heterogeneity
Source: PLoS One. 2012 Nov 30;7(11):e50715. doi: 10.1371/journal.pone.0050715 (PMC3511357; doi:10.1371/journal.pone.0050715)
Supplement: Methods S1 — Supplemental information. (DOCX) [file pone.0050715.s008.docx]

**SUPPLEMENTAL INFORMATION**

**Stochastic kinetics of Nanog expression**

The dynamics of Nanog –depicted by the rate r_2_(N)– was modeled according to the network shown below. Such network structures are routinely implemented for modeling gene transcription and translation processes [[1](#_ENREF_1),[2](#_ENREF_2)]. The *NANOG* gene promoter switches between an active (A) or repressed (R) state with transition constants k_on_ and k_off_. The gene is transcribed into mRNA (M) from either state with rates S_A_ and S_R_ and further translated into NANOG protein (N) with rate S_P_. The degradation rate constants of the mRNA and protein are δ_M_ and δ_N_, respectively. First-order kinetics was assumed for all steps.


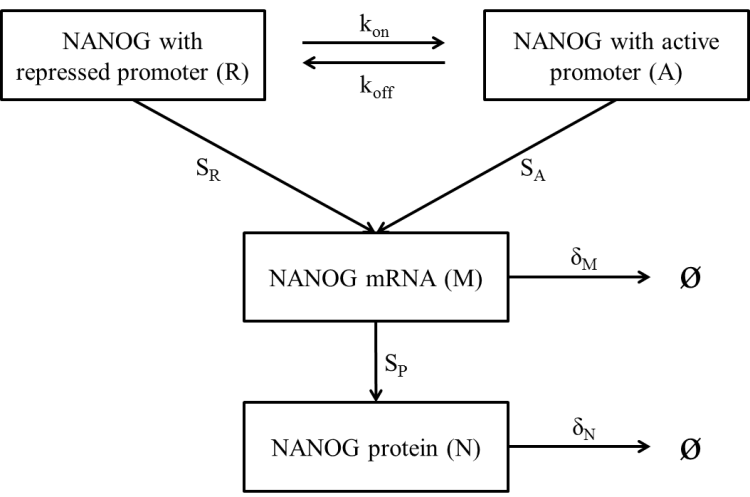


Figure SA1. Schematic of the single gene expression model utilized in this study.

$\frac{dM}{dt} = \frac{k_{on}}{k_{on}+k_{off}}\cdot S_{A}+\frac{k_{off}}{k_{on}+k_{off}}\cdot S_{R}-\delta_{M}\cdot M+\varepsilon(t)$ (Equation S1)

$\frac{dN}{dt} =S_{p}\cdot M-\delta_{N}\cdot N$ (Equation S2)

Equation S1 is stochastic with ε(t) being the Gaussian white noise with mean at zero and standard deviation σ. The noise term is employed at the transcription level and stochasticity propagates to the translation of NANOG [[3](#_ENREF_3)].

Since the PBE model in this study takes into account the partition of intracellular components at cell division, the profile of NANOG is affected by both molecular reaction noise and partitioning at cellular level. However, synthesis and degradation of mRNA are very rapid events compared to the timescale of mitosis-induced partitioning. So here we assume that the partitioning effect does not have effect on mRNA levels during the interval between successive divisions of the same cell. Then

$M=\frac{k_{on}\cdot S_{A}+k_{off}\cdot S_{R}}{(k_{on}+k_{off})\cdot\delta_{M}}+\frac{\varepsilon\left(t\right)}{\delta_{M}}$ (Equation S3)

$\frac{dN}{dt} =\frac{k_{on}\cdot S_{A}+k_{off}\cdot S_{R}}{(k_{on}+k_{off})\cdot\delta_{M}}\cdot S_{p}-\delta_{N}\cdot N+\frac{S_{p}}{\delta_{M}}\cdot\varepsilon\left(t\right)$ (Equation S4)

The expression for NANOG protein dynamics can be simplified:

$\frac{dN}{dt} = a-d\cdot N+\delta\cdot\varepsilon\left(t\right)$ (Equation S5)

where $a=\frac{k_{on}\cdot S_{A}+k_{off}\cdot S_{R}}{(k_{on}+k_{off})\cdot\delta_{M}}\cdot S_{p}$, $d=\delta_{M}$, $\delta=\frac{S_{p}}{\delta_{M}}\cdot\sigma$ and ε(t) is a Gaussian white noise with mean μ=0 and standard deviation σ=1. Thus, parameter δ could be considered as the intensity of noise at the protein level.

**Supplemental Methods**

1. **Differentiation of human embryonic stem cells**

**1.1 Endoderm differentiation**

Differentiation of hESCs in definitive endoderm (DE) was induced as described [[4](#_ENREF_4)]. Briefly, cells were seeded on dishes coated with Matrigel (BD Biosciences) with mTeSR medium (StemCell Technologies). When cells reached 80% confluence, differentiation was carried out in RPMI (GIBCO, Grand Island, NY) supplemented with 100 ng/ml activin A (R&D Systems) from days 0 to 4. On day 1, 0.2% Knockout Serum Replacer (KSR) (GIBCO) was added in the differentiation medium. At day 2 the concentration of KSR was adjusted to 2% and maintained at that level. Control samples were treated with the same basal medium but without activin A.

For further differentiation to posterior foregut (PF), hESC-derived DE cells were treated as described [[5](#_ENREF_5)] with modifications [[6](#_ENREF_6)]. After induction of hESCs into DE stage, hESCs were further directed into primitive gut tube (PGT) for 4 days treated by 2% KSR in RPMI supplemented with 50 ng/ml keratinocyte growth factor (KGF), 2 mM retinoic acid (RA) and 200 ng/ml epidermal growth factor (EGF) (all growth factors from R&D systems, Minneapolis, MN). After a 4-day induction into primitive gut tube, cells were further directed to the PF stage. Differentiation medium contained KGF, RA and EGF as in the PGT stage but in DMEM/F12 with 1×N2 and 1×B27 (GIBCO) as well as 0.5% bovine serum albumin (BioFX Laboratories, Owing Mills, MD). Control samples were treated with the same basal medium but without any growth factor.

**1.2 Mesoderm differentiation**

Differentiation into mesoderm was induced by activin A and BMP4 for five days [[7](#_ENREF_7)]. Human ESCs were seeded on Matrigel-coated dishes with mTeSR medium. When cells reached 80% confluence, differentiation was carried out in RPMI medium supplemented with 100 ng/ml activin A at day 0. At day 1, RPMI supplemented with 0.2% FBS (PAA Laboratories, Dartmouth, MA), 10 ng/ml activin A and 10 ng/ml BMP4 (R&D Systems) was added to the dishes. From day 2 to day 4, RPMI supplemented with 2% FBS, 10 ng/ml activin A and 10 ng/ml BMP4 was added to the dishes. Control samples were treated with the same basal medium but without activin A and BMP4.

- 1. **Ectoderm differentiation**

For ectoderm differentiation, neural induction (NIM) and neural proliferation media (NPM) were used [[8](#_ENREF_8)]. NIM contained DMEM/F12:Neurobasal medium (1:1), 1×N2 supplement, 1×B27 supplement without vitamin A (all from GIBCO) and 2 mM Glutamax (Mediatech, Herndon, VA). NPM contained DMEM/F12:Neurobasal(1:1), 0.5×N2 supplement, 0.5×B27 supplement, 2 mM Glutamax and 20 ng/mL FGF2 (R&D Systems).

NIM was added when hESCs were over 90% confluent. The next day hESC colonies were treated with collagenase IV (GIBCO) and seeded in low-attachment dishes (BD Biosciences) to form aggregates. The cells were cultured with NIM for 6 days. Then the culture medium was switched to NPM for another 7 days. After a 7-day culture with NPM, aggregates were removed for RNA isolation. Alternatively, cells were transferred onto Matrigel-coated slides for immunocytochemistry. Control samples consisted of hESCs treated with 0.2% KSR in RPMI for 4 days.

1. **RNA extraction, RT-PCR and quantitative PCR**

Total RNA was isolated from cells using Trizol (Invitrogen) according to the manufacturer’s instructions. Reverse transcription was performed using the ImProm-II reverse transcriptase (Promega) with 1 μg of total RNA and 250 ng random primers or oligo(dT)_12-18_ primers at 42^o^C for 60 min. PCR runs were carried out with the resulting cDNA for 30-35 cycles at an annealing temperature of 58-60^o^C depending on the primer set. Primer sequences are shown in Table S1.

For relative quantification of gene expression, standard real-time polymerase chain reactions were performed on an CFX96 quantitative PCR (qPCR) machine (Bio-Rad, Hercules, CA) as described previously [[4](#_ENREF_4)]. The iQ SYBR Green qPCR mix (Bio-Rad) was used with the following reaction conditions: Denaturation and polymerase activation at 95^o^C for 2 min; amplification for 40 cycles at 95^o^C for 15 sec, 58^o^C or 60^o^C for 30 sec and 72^o^C for 1 min. All reactions were run in triplicates on samples from at least 3 experiments. Amplification specificity was verified by a melting curve method. Relative gene expression was calculated by normalizing to the expression of endogenous β-actin, using the ΔΔC_T_ method [[9](#_ENREF_9)]. The threshold cycle (C_T_) for β-actin did not vary under different experimental conditions when equal amounts of RNA were used.

1. **Immunocytochemistry**

Cells were stained for immunofluorescence as described previously [[4](#_ENREF_4)]. Briefly, cells were washed in PBS and fixed with 4% paraformaldehyde (Sigma-Aldrich) in PBS (pH 7.4) for 20 min at room temperature. After washing with PBS, cells were permeabilized with 0.1% Triton X-100 (Mallinckrodt Baker, Phillipsburg, NJ) in PBS for 15 min and then blocked with 1% normal donkey serum (NDS; Jackson ImmunoResearch Laboratories, West Grove, PA) in PBS for 1 hr. Incubation was carried out overnight at 4^o^C with a primary antibody (see list below). After three washes with PBS for 5 min, secondary antibodies were applied for 1 hr at room temperature. Corresponding secondary antibodies conjugated with Dylight 488 or 549 (Jackson ImmunoResearch Laboratories; see list below) were used. Micrographs were acquired with either a Zeiss Observer Z1 or D1 microscope equipped with an Axiocam MRm camera (Carl Zeiss, Thornwood, NY).

The following antibodies were utilized for immunocytochemistry:

Primary antibodies:

Sox 17: Polyclonal antibody, goat, R&D Systems, cat. no.: AF1924

Foxa2: Polyclonal antibody, rabbit, Cell Signaling Technology, cat. no.: 3143S

Meox1: Polyclonal antibody, rabbit, Novus Biologicals, cat. no.: NBP1-92134

Flk1: Monoclonal antibody, mouse, Abcam, cat. no.: ab9530

Nestin: Monoclonal antibody, mouse, R&D systems, cat. no.: MAB1259

β-III-tubulin: Polyclonal antibody, rabbit, Sigma-Aldrich, cat. no.: T3952

Secondary antibodies:

Donkey anti-rabbit IgG H&L (DyLight 488), Jackson Immunoresearch, cat. no.: 711-485-152

Donkey anti-mouse IgG H&L (DyLight 549), Jackson Immunoresearch, cat. no.: 715-505-151

Donkey anti-goat IgG H&L (DyLight 549), Jackson Immunoresearch, cat .no.: 705-505-147

**REFERENCES**

1. Kaern M, Elston TC, Blake WJ, Collins JJ (2005) Stochasticity in gene expression: from theories to phenotypes. Nat Rev Genet 6: 451-464.

2. Wang L, Walker BL, Iannaccone S, Bhatt D, Kennedy PJ, et al. (2009) Bistable switches control memory and plasticity in cellular differentiation. Proc Natl Acad Sci USA 106: 6638-6643.

3. Rao CV, Wolf DM, Arkin AP (2002) Control, exploitation and tolerance of intracellular noise. Nature 420: 231-237.

4. Lock LT, Tzanakakis ES (2009) Expansion and differentiation of human embryonic stem cells to endoderm progeny in a microcarrier stirred-suspension culture. Tissue Eng Part A 15: 2051-2063.

5. Kroon E, Martinson LA, Kadoya K, Bang AG, Kelly OG, et al. (2008) Pancreatic endoderm derived from human embryonic stem cells generates glucose-responsive insulin-secreting cells in vivo. Nat Biotechnol 26: 443-452.

6. Lock LT (2011) PhD Thesis: Expansion and directed differentiation of human pluripotent stem cells to insulin-producing cells in a stirred-suspension microcarrier system. United States -- New York: State University of New York at Buffalo. 158 p.

7. Jing D (2010) PhD Thesis: Investigation of the cardiogenic differentiation of human pluripotent stem cells in static cultures and stirred-suspension bioreactors. United States -- New York: State University of New York at Buffalo. 188 p.

8. Nat R, Nilbratt M, Narkilahti S, Winblad B, Hovatta O, et al. (2007) Neurogenic neuroepithelial and radial glial cells generated from six human embryonic stem cell lines in serum-free suspension and adherent cultures. Glia 55: 385-399.

9. Livak KJ, Schmittgen TD (2001) Analysis of relative gene expression data using real-time quantitative PCR and the 2(-Delta Delta C(T)) Method. Methods (San Diego, Calif 25: 402-408.
